# Supplementary material for: Sex differences in health-related quality of life trajectories following myocardial infarction: national longitudinal cohort study
Source: BMJ Open. 2022 Nov 8;12(11):e062508. doi: 10.1136/bmjopen-2022-062508 (PMC9644325; doi:10.1136/bmjopen-2022-062508)
Supplement: Supplementary data [file bmjopen-2022-062508supp001.pdf]

SUPPLEMENTARY MATERIAL

Title: Sex differences in health-related quality of life trajectories following acute myocardial infarction: National longitudinal cohort study.

Authors

T.B. Dondo (PhD), T. Munyombwe (PhD), M. Hall (PhD), B. Hurdus (MBBS), A.Soloveva (PhD)[1,2], G. Oliver (Patient), S. Aktaa (MD), RM West (DPhil), A.S. Hall (FRCP), C.P. Gale (PhD FRCP).

Section 1: Supplementary methods

Propensity score analysis

A non-parsimonious multivariable logistic regression model was used to derive propensity scores (PS) (probability of being a man or a woman, conditional on observed patient baseline covariates) to weight the data balancing out systematic differences. The model was adjusted for patient baseline characteristics; AMI phenotype (STEMI vs. NSTEMI), age, body mass index (BMI), index of multiple deprivation (IMD) score, smoking status (never vs. current or ex-smoker), family history of coronary heart disease (CHD), previous angina, history of diabetes mellitus, hypertension, heart failure, peripheral vascular disease, cerebrovascular disease, chronic renal failure, chronic obstructive pulmonary disease (COPD) or asthma, hypercholesterolemia, previous percutaneous coronary intervention (PCI), previous coronary artery bypass graft (CABG) surgery, previous AMI, coronary intervention, hospital discharge medications (aspirin, β blockers, statins, ACEi/ARB and $P2Y_{12}$ inhibitors) and referral for cardiac rehabilitation. In order to assess whether the weights constructed from the propensity score model balanced the covariates between men and women, standardised differences were derived, a perfectly balanced covariate has a standardised difference of zero. Violation of the overlap assumption was assessed using an overlap plot and by summarising the estimated probabilities of sex. Supplemental Figure 1 illustrates the results of the assessment of the overlap assumption and shows that the minimum propensity score for each level was sufficiently greater than zero and that the maximum propensity score for each level was sufficiently less than 1, thus the assumption was not violated. The area under the curve for the propensity score model was 0.64, which indicated moderate discrimination for the model.

Supplementary Figure 1. Overlap assumption assessment plots (A) and Distribution of propensity scores across comparison groups (B).

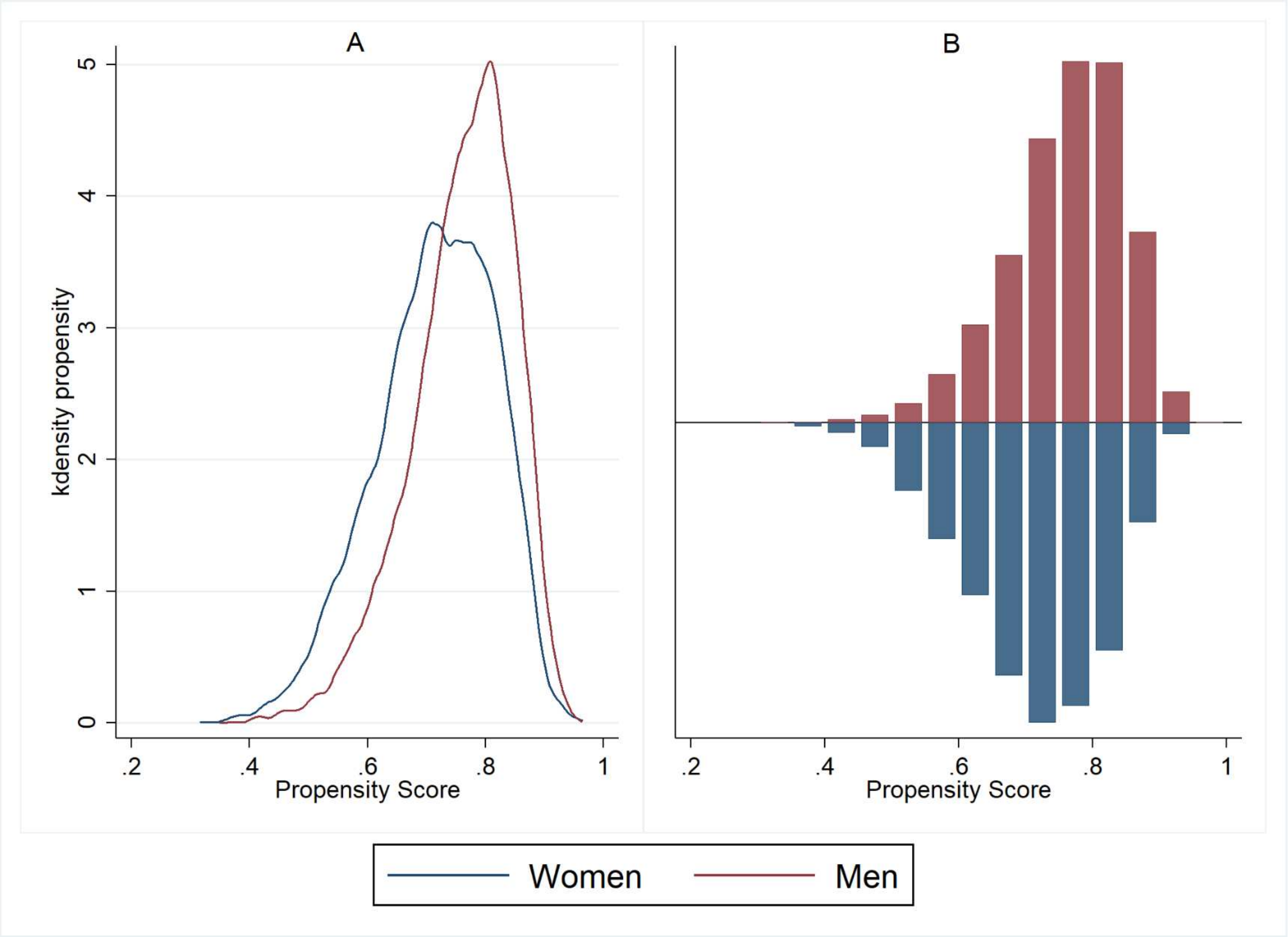

Section 2: Supplementary methods

Handling missing data in baseline characteristics

Missing data were imputed using multiple imputations by chained equations. Ten imputed datasets were derived from 20 iterations. Data were imputed for missing baseline characteristics and not follow-up outcome data. A default imputation (missing data default imputed to "NO") strategy based on clinical expert opinion was implemented for cardiovascular history, cardiovascular risk factors, and categorical treatment variables. The imputation strategy applied is summarised in Supplemental Table 1.

Supplementary Table 1. Imputation Strategy

Variable	Variable Type	Missing n (%)	Imputation Method
Sex	Binary	15 (0.16)	Predictor/ Auxiliary
Age	Continuous	19 (0.20)	Predictive mean matching
Family history of CHD	Binary	1,473 (15.40)	Predictor/ Auxiliary and Default imputed
Previous PCI	Binary	510 (5.33)	Predictor/ Auxiliary and Default imputed
Previous CABG	Binary	496 (5.19)	Predictor/ Auxiliary and Default imputed
Overallimdscore	Continuous	5,260 (55.00)	Predictive mean matching
BMI	Continuous	3,374 (35.27)	Predictive mean matching
Cardiac rehabilitation referral at hospital discharge	Binary	609 (6.37)	Predictor/ Auxiliary and Default imputed
Previous AMI	Binary	486 (5.08)	Predictor/ Auxiliary and Default imputed
Previous Angina	Binary	493 (5.15)	Predictor/ Auxiliary and Default imputed
Hypertension	Binary	487 (5.09)	Predictor/ Auxiliary and Default imputed
Hypercholesterolaemia	Binary	567 (5.93)	Predictor/ Auxiliary and Default imputed

Variable	Variable Type	Missing n (%)	Imputation Method
Peripheral Vascular Disease	Binary	626 (6.54)	Predictor/ Auxiliary and Default imputed
Cerebrovascular Disease	Binary	496 (5.19)	Predictor/ Auxiliary and Default imputed
Asthma or COPD	Binary	506 (5.29)	Predictor/ Auxiliary and Default imputed
Chronic Renal Failure	Binary	499 (5.22)	Predictor/ Auxiliary and Default imputed
Diabetes	Binary	330 (3.45)	Predictor/ Auxiliary and Default imputed
Reinfarction	Binary	510 (5.33)	Predictor/ Auxiliary and Default imputed
EQ-5D at admission	Continuous	366 (3.83)	Predictor/ Auxiliary
EQVAS at admission	Continuous	303 (3.17)	Predictor/ Auxiliary
Mobility	Binary	234 (2.45)	Predictor/ Auxiliary
Self-care	Binary	250 (2.61)	Predictor/ Auxiliary
Usual activities	Binary	300 (3.14)	Predictor/ Auxiliary
Pain/discomfort	Binary	250 (2.61)	Predictor/ Auxiliary
Anxiety/depression	Binary	252 (2.63)	Predictor/ Auxiliary

Abbreviations: CABG, coronary artery bypass graft; PCI, percutaneous coronary intervention; MI, Myocardial Infarction; COPD, chronic obstructive pulmonary disease; BMI, body mass index; CHD, coronary heart disease.

Supplementary Table 2. Patient baseline characteristics (patients with complete follow-up data vs patients with missing follow-up data at one or time points).

Variables	Patients with complete follow up data at all-time points (n= 3,413)	Patients with missing follow-up data at one or time points (n= 6,138)	P value
NSTEMI, n. (%)	2,051 (60.1)	3,597 (58.6)	0.155
Age, mean (SD), yr.	65.9 (10.6)	63.1 (12.5)	<0.001
White ethnicity, n. (%)	2,915 (98.7)	5,211 (96.5)	<0.001
IMD, median (IQR)	16.3 (10.2-27.1)	20.6 (11.9-34.0)	<0.001
BMI, mean(SD), kg/ m^2	27.4 (24.9-30.8)	28.1 (25.1-31.6)	<0.001
Previous angina, n. (%)	619 (19.0)	1,171 (20.2)	0.193
Diabetes, n. (%)	506 (15.4)	1,207 (20.3)	<0.001
Hypertension, n. (%)	1,486 (45.7)	2,588 (44.5)	0.303
Heart failure, n. (%)	62 (1.9)	150 (2.6)	0.042
Peripheral vascular disease, n. (%)	97 (3.1)	220 (3.8)	0.056
Cerebrovascular disease, n. (%)	136 (4.2)	292 (5.0)	0.069
Chronical renal failure, n. (%)	76 (2.3)	213 (3.7)	<0.001
COPD, n. (%)	374 (11.5)	792 (13.7)	0.004
Smoker and ex-smoker, n. (%)	2,050 (62.0)	4,192 (70.1)	<0.001
CABG surgery, n. (%)	227 (7.0)	414 (7.1)	0.826
Previous PCI, n. (%)	300 (9.3)	597 (10.3)	0.119
Previous AMI, n. (%)	485 (14.9)	1,035 (17.8)	<0.001
Cardiac rehabilitation† (n=8,700), n. (%)	3,049 (97.8)	5,448 (97.6)	0.681
Coronary intervention† (n=7,261), n. (%)	1,563 (60.3)	2,765 (59.2)	0.353
**Discharge medications†**			

Variables	Patients with complete follow up data at all-time points (n= 3,413)	Patients with missing follow-up data at one or time points (n= 6,138)	P value
Beta-blocker (n=7,708), n (%)	2,741 (98.4)	4,838 (98.3)	0.764
ACEi or ARB inhibitor (n=7,786), n. (%)	2,742 (97.8)	4,856 (97.5)	0.467
Statin (n=8,203), n. (%)	2,911 (99.3)	5,216 (99.0)	0.214
Aspirin (n=8,191), n. (%)	2,920 (99.3)	5,213 (99.3)	0.822
$P2Y_{12}$ inhibitors (n=5,005), n. (%)	1,828 (97.4)	3,041 (97.2)	0.593

Abbreviations: ACEi – angiotensin-converting enzyme inhibitor; ACS – Acute coronary syndrome; ARBs – Angiotensin receptor blocker, IMD indicates Index of Multiple Deprivation; CABG, coronary artery bypass graft; PCI, percutaneous coronary intervention; MI, Myocardial Infarction; COPD, chronic obstructive pulmonary disease; BMI, body mass index; NSTEMI, non ST-elevation myocardial infarction.

Section 3: Supplementary Tables

The balance check results are summarised in Supplemental Table 3, which shows that the standardised differences for variables in the weighted data were close to zero. The diagnostic assessments suggest that weighting by the inverse probability of sex created a sample in which the distributions of the covariates were similar between men and women.

Supplementary Table 3. Covariate balance across men and women after weighting on the propensity score

Variable	Mean in men	Mean in women	Standardized difference
Age	62.28	65.62	-0.285
Deprivation (IMD score)	22.55	24.68	-0.135
BMI	28.46	29.05	-0.108
Smoker ever	0.69	0.64	0.118
Final diagnosis	0.44	0.41	0.058
Family history of CHD	0.40	0.40	0.003
Previous PCI	0.07	0.06	0.023
Previous CABG	0.06	0.04	0.092
Previous MI	0.13	0.09	0.132
Hypertension	0.43	0.53	-0.201
Hypercholesterolaemia	0.34	0.34	-0.008
PVD	0.03	0.04	-0.049
CVSD	0.04	0.04	0.038
COPD	0.11	0.16	-0.126
Chronic renal failure	0.02	0.04	-0.106
Chronic cardiac failure	0.01	0.01	0.033
Diabetes	0.15	0.17	-0.046
Discharge medications			
Aspirin	0.89	0.87	0.050
β blockers	0.83	0.83	-0.002
Statin	0.89	0.88	0.036
ACEi/ARBs	0.85	0.83	0.056
$P2Y_{12}$ inhibitors	0.78	0.77	0.015

Coronary intervention	0.52	0.45	0.139
Cardiac rehabilitation	0.93	0.93	0.005

Abbreviations: ACEi – angiotensin-converting enzyme inhibitor; ACS – Acute coronary syndrome; ARBs – Angiotensin receptor blocker, IMD indicates Index of Multiple Deprivation; CABG, coronary artery bypass graft; PCI, percutaneous coronary intervention; MI, Myocardial Infarction; COPD, chronic obstructive pulmonary disease; CVSD cerebrovascular disease; BMI, body mass index.
