# Supplementary material for: Sex differences in health-related quality of life trajectories following myocardial infarction: national longitudinal cohort study
Source: BMJ Open. 2022 Nov 8;12(11):e062508. doi: 10.1136/bmjopen-2022-062508 (PMC9644325; doi:10.1136/bmjopen-2022-062508)
Supplement: Author's manuscript [file bmjopen-2022-062508.draft_revisions.pdf]

BMJ Open is committed to open peer review. As part of this commitment we make the peer review history of every article we publish publicly available.

When an article is published we post the peer reviewers' comments and the authors' responses online. We also post the versions of the paper that were used during peer review. These are the versions that the peer review comments apply to.

The versions of the paper that follow are the versions that were submitted during the peer review process. They are not the versions of record or the final published versions. They should not be cited or distributed as the published version of this manuscript.

BMJ Open is an open access journal and the full, final, typeset and author-corrected version of record of the manuscript is available on our site with no access controls, subscription charges or pay-per-view fees (http://bmjopen.bmj.com).

If you have any questions on BMJ Open's open peer review process please email info.bmjopen@bmj.com

BMJ Open

Sex differences in health-related quality of life trajectories following myocardial infarction: National longitudinal cohort study.

Journal:	*BMJ Open*
Manuscript ID	bmjopen-2022-062508
Article Type:	Original research
Date Submitted by the Author:	02-Mar-2022
Complete List of Authors:	Dondo, Tatendashe; University of Leeds, Leeds Institute of Cardiovascular and Metabolic Medicine; University of Leeds, Leeds Institute for Data Analytics<br>Munyombwe, T; University of Leeds, Leeds Institute of Cardiovascular and Metabolic Medicine; University of Leeds, Leeds Institute for Data Analytics<br>Hall, Marlous ; University of Leeds, Leeds Institute of Cardiovascular and Metabolic Medicine ; University of Leeds, Leeds Institute for Data Analytics<br>Hurdus, B; University of Leeds, Leeds institute for data analytics; Leeds Teaching Hospitals NHS Trust, Department of Cardiology<br>Soloveva, A.; University of Leeds, Leeds Institute of Cardiovascular and Metabolic Medicine; University of Leeds, Leeds Institute for Data Analytics<br>Oliver, Gerard; West yorkshire<br>Aktaa, Suleman; University of Leeds, Leeds Institute of Cardiovascular and Metabolic Medicine ; Leeds General Infirmary, Department of Cardiology Leeds General Infirmary<br>West, Robert; University of Leeds, Leeds Institute of Health Sciences<br>Hall, Alistair; Leeds General Infirmary Department of Cardiology, Cardiology Department; University of Leeds, Leeds Institute of Cardiovascular and Metabolic Medicine<br>Gale, Chris; University of Leeds, Leeds Institute of Cardiovascular and Metabolic Medicine ; Leeds General Infirmary, Department of Cardiology Leeds General Infirmary
Keywords:	Myocardial infarction < CARDIOLOGY, Epidemiology < TROPICAL MEDICINE, Coronary heart disease < CARDIOLOGY

SCHOLARONE™
Manuscripts

I, the Submitting Author has the right to grant and does grant on behalf of all authors of the Work (as defined in the below author licence), an exclusive licence and/or a non-exclusive licence for contributions from authors who are: i) UK Crown employees; ii) where BMJ has agreed a CC-BY licence shall apply, and/or iii) in accordance with the terms applicable for US Federal Government officers or employees acting as part of their official duties; on a worldwide, perpetual, irrevocable, royalty-free basis to BMJ Publishing Group Ltd ("BMJ") its licensees and where the relevant Journal is co-owned by BMJ to the co-owners of the Journal, to publish the Work in this journal and any other BMJ products and to exploit all rights, as set out in our licence.

The Submitting Author accepts and understands that any supply made under these terms is made by BMJ to the Submitting Author unless you are acting as an employee on behalf of your employer or a postgraduate student of an affiliated institution which is paying any applicable article publishing charge ("APC") for Open Access articles. Where the Submitting Author wishes to make the Work available on an Open Access basis (and intends to pay the relevant APC), the terms of reuse of such Open Access shall be governed by a Creative Commons licence – details of these licences and which Creative Commons licence will apply to this Work are set out in our licence referred to above.

Other than as permitted in any relevant BMJ Author's Self Archiving Policies, I confirm this Work has not been accepted for publication elsewhere, is not being considered for publication elsewhere and does not duplicate material already published. I confirm all authors consent to publication of this Work and authorise the granting of this licence.

For peer review only

Sex differences in health-related quality of life trajectories following myocardial infarction: National longitudinal cohort study.

Authors

T.B. Dondo (PhD)[1,2], T. Munyombwe (PhD)[1,2], M. Hall (PhD)[1,2], B. Hurdus (MBBS)[1,2,3], A.Soloveva (PhD)[1,2], G. Oliver (Patient)[4], S. Aktaa (MD)[1,2,3] ,R.M. West (DPhil)[5], A.S. Hall (FRCP)[1,3], C.P. Gale (PhD FRCP)[1,2,3].

Affiliations

[1]Leeds Institute of Cardiovascular and Metabolic Medicine, University of Leeds, Leeds, UK

[2] Leeds Institute for Data Analytics, University of Leeds, Leeds, United Kingdom

[3] Department of Cardiology Leeds General Infirmary, Leeds, UK

[4]Patient representative, Lancashire, United Kingdom

[5]Leeds Institute of Health Sciences, University of Leeds, Leeds, United Kingdom

Correspondence

Dr Tatendashe Bernadette Dondo

Leeds Institute of Cardiovascular and Metabolic Medicine,

MRC Bioinformatics Centre, Level 11, Worsley building, Clarendon way,

Leeds, LS2 9JT

Email: t.b.dondo@leeds.ac.uk

Tel: 0044 (0)113 343 8905

Twitter @bernadetteTDond

For peer review only - http://bmjopen.bmj.com/site/about/guidelines.xhtml

Abstract

Objectives: To investigate sex-based differences in baseline values and longitudinal trajectories of health-related quality of life (HRQoL) in a large cohort of myocardial infarction (MI) survivors after adjusting for other important factors.

Design: Longitudinal cohort study

Setting: Population-based longitudinal study the Evaluation of the Methods and Management of Acute Coronary Events (EMMACE) study linked with national cardiovascular registry. Data was collected from 77 hospitals in England between 1 November 2011 and 24 June 2015.

Participants: 9,551 patients with MI. Patients were eligible for the study if they were ≥18 years of age.

Primary and secondary outcome measures: HRQoL was measured by EuroQol five-dimension (EQ-5D, EQ VAS) survey at baseline, 1, 6 and 12 months after discharge. Multi-level linear and logistic regression models coupled with inverse probability weighted propensity scoring were used to evaluate sex differences in HRQoL following MI.

Results: Of the 9,551 patients with MI and complete data on sex, 25.1% (2,397) were women. At baseline, women reported lower HRQoL (EQ VAS [mean (SD) 59.8 (20.4) vs. 64.5 (20.9)] [median (IQR) 60.00 (50.00 to 75.00) vs. 70.00 (50.00 to 80.00)]) (EQ-5D [mean (SD) 0.66 (0.31) vs. 0.74 (0.28)] [median (IQR) 0.73 (0.52 to 0.85) vs. 0.81 (0.62 to 1.00)]) and were more likely to report problems in each HRQoL domain compared with men. In the covariate balanced and adjusted multi-level models sex differences in HRQoL persisted during follow-up, with lower EQ VAS and EQ-5D scores in women compared with men (adjusted EQ VAS model sex coefficient: -4.41, 95% CI -5.16 to -3.66 and adjusted EQ-5D model sex coefficient: -0.07, 95% CI -0.08 to -0.06).

For peer review only - http://bmjopen.bmj.com/site/about/guidelines.xhtml

Conclusions: Women have lower HRQoL compared with men at baseline and during 12 months follow-up after MI. Tailored interventions for women following an MI could improve their quality of life.

Clinical Trial Registration: ClinicalTrials.gov (NCT04598048, NCT01808027, NCT01819103)

Keywords: Quality of life, Women, Myocardial infarction, Epidemiology, Acute coronary syndrome

For peer review only

For peer review only - http://bmjopen.bmj.com/site/about/guidelines.xhtml

Strengths and limitations of this study

- Data source is linked nationwide longitudinal HRQoL data which minimises selection bias and increases generalisability.
- An inverse weighted propensity scoring approach was applied to weight data and balance out systematic differences based on observed covariates to minimise inherent bias.
- Used generic quality of life metric rather than a disease-specific one to measure HRQoL following MI.
- Potential selection bias due to loss to follow-up.

For peer review only

Introduction

Recent decades were characterized by significant decline of mortality in myocardial infarction (MI). Consequently, health-related quality of life (HRQoL) following MI emerged as another important indicator of patient care. HRQoL represents patients' perspective of their health state but also serves as an important clinical risk marker and treatment target given lower HRQoL in MI survivors is independently associated with increased risk of death[1].

Emerging evidence points to the significant sex-based differences in MI population that may also account for HRQoL differences. The exact explanation for this phenomenon remains uncertain, but distinct clinical presentation and etiology of MI, higher age and comorbidity burden, less frequent invasive therapeutic approach, higher rehospitalisation rates and long-term mortality had been consistently shown in women compared with men [2-5]. Importantly, these differences in characteristics and treatment strategies may impact not only HRQoL at the time of the acute event but also its trajectories over time. Previous studies that demonstrated lower HRQoL scores in women compared with men were either small [6-10] or focused on a selected subgroup of MI patients [11 12] thus were unable to adjust for multiple confounding factors or answer the question of independent sex differences in a heterogeneous MI population. Moreover, only a few contemporary studies explored longitudinal HRQoL estimates depending on sex [6 8-10 12 13] thus an appropriate time for the subsequent assessment of HRQoL remains unknown. Knowledge of such gender-based disparities in HRQoL is important as it could highlight the need for strategies to improve the health status of women following MI. Furthermore, understanding the differences in the HRQoL domains may provide an opportunity to identify the components of patient-reported health that need particularly higher attention and clinical counseling. Using a large nationwide longitudinal

For peer review only - http://bmjopen.bmj.com/site/about/guidelines.xhtml

cohort study of consecutive patients hospitalised with acute coronary syndrome (ACS) we aimed to investigate sex differences in HRQoL in MI survivals, the longitudinal trajectories of HRQoL over a 12-month period, and determine to what extent sex itself might explain the differences in HRQoL when accounting for other important factors.

Methods

Design and setting

Linked data from the Evaluation of the Methods and Management of Acute Coronary Events (EMMACE 3 and 4)[14] and Myocardial Ischaemia National Audit Project (MINAP)[15] were used for the analyses. The EMMACE studies are multi-centre nationwide longitudinal cohort studies of patients hospitalised with ACS. Patients were eligible for the study if they were ≥18 years of age. HRQoL data for MI survivors from 77 hospitals in England between 1 November 2011 and 24 June 2015 were collected at hospital admission (baseline), and longitudinally at 1, 6 and 12 months via questionnaires. Patients were consented for data linkage with MINAP to obtain information on the type of MI, baseline co-morbidities and in-hospital treatments. Fifteen participants (0.2%) had missing sex data and were excluded from the study. Of the 9,551 participants 35.7% (3,413) completed and returned the questionnaires at all-time points data was collected.

Assessment of HRQoL

EuroQol five dimension (EQ-5D-3L) questionnaire was used to collect HRQoL data[16]. The EQ-5D questionnaire consists of questions covering five health domains, which include mobility, self-care, usual activities, pain/ discomfort and anxiety/ depression. An EQ-5D single score is derived based on the questions taking into account societal preference weights [17]. The EQ-5D index score ranges from −0.5 to 1, with scores less than 0 indicating states ‘worse than death’, 0 indicating no quality of life or ‘death’ and 1 indicating full health and

For peer review only

For peer review only - http://bmjopen.bmj.com/site/about/guidelines.xhtml

therefore no problems in any domain. The index score has been standardised to the UK population and validity of the questionnaire in MI patients has been determined[16-18]. The questionnaire also has a visual analogue scale (EQ VAS) that allows participants to rate their current health state. The EQ VAS score ranges from 0 to 100 with 0 denoting the worst imaginable health state and 100 the best imaginable health state. A difference in the score of 7 for EQ VAS and 0.05 for EQ-5D is regarded as the minimal clinically important difference (MCID)[19].

Statistical analyses

Differences in baseline characteristics for men and women were described using frequencies and proportions for categorical data, means and standard deviations (SD) for normally distributed continuous data and medians and interquartile ranges (IQR) for non-normally distributed data. Multi-level linear regression was used to assess sex differences in HRQoL (EQ-5D and EQ VAS scores) in MI survivors. As the HRQoL data consisted of repeated measures nested within individuals and individuals nested within hospitals, the multi-level approach was implemented. Inverse probability weighted propensity scoring was used to weight the data and balance out systematic differences in baseline characteristics between men and women to minimise selection bias (See Supplementary methods section 1, which gives further detail of the methods). The primary outcomes of the study were the EQ-5D and EQ VAS scores – with further subgroup analyses conducted for each of the EQ-5D domains (mobility, self-care, usual activities, pain/discomfort and anxiety/ depression) using multi-level logistic regression models. The domains are recorded as three level variables, however, for this study they were treated as binary variables, ‘some problems’ and ‘extreme problems’ levels vs. ‘no problems’. To mitigate residual confounding the multi-level linear and logistic regression models were adjusted for covariates which included aspirin, β-blockers, statins, angiotensin-converting enzyme (ACE) inhibitor or angiotensin receptor blocker (ARB) and

For peer review only - http://bmjopen.bmj.com/site/about/guidelines.xhtml

$P2Y_{12}$ inhibitors prescription at hospital discharge, type of MI, enrolment into cardiac rehabilitation, coronary intervention, body mass index (BMI), previous MI, age, index of multiple deprivation (IMD) score, previous coronary artery bypass graft (CABG) surgery, smoking status, previous percutaneous coronary intervention (PCI), family history of coronary heart disease (CHD), peripheral vascular disease, hypercholesterolemia, previous angina, chronic obstructive pulmonary disease (COPD) or asthma, diabetes mellitus, chronic renal failure, hypertension, cerebrovascular disease and heart failure. Effect sizes were estimated evaluating changes in HRQoL over time, i.e. from time of hospitalisation with MI (baseline) to 12 months post hospital discharge. An interaction term of time and sex was added to the models to test if there were significant sex differences in rate of improvement in HRQoL following AMI. The models were fitted on the weighted balanced data.

Multiple imputation by chained equations[20] was used to impute missing data for the following variables: age, IMD score and BMI (See Supplementary Table 1, section 2, which gives detail of the imputation strategy used). Based on clinical expert opinion select binary treatment and medical history variables were imputed to 'no' if missing (See Supplementary Table 1, section 2, which gives detail of the imputation strategy used)[20]. Rubin's rules [21] were used to pool the results estimates and generate 95% confidence intervals. On non-weighted data predictors of change in HRQoL were explored by sequentially adding covariates (baseline HRQoL patient reported measures and patient baseline characteristics) to the bivariate multi-level linear regression model with sex only. Covariates which attenuated the sex differences in change in HRQoL observed were considered as predictors. Analysis were performed using Stata MP64 version 14 (StataCorp,www.stata.com) and R version 3.1.2. P-values <0.05 were considered statistically significant.

For peer review only - http://bmjopen.bmj.com/site/about/guidelines.xhtml

200 **Patient and public involvement**

201 The Leeds Teaching Hospitals NHS Trust Cardiovascular Patient and Public Involvement
202 group was involved in the project design. We also worked closely with a patient (GO) outside
203 the group for the interpretation of the research findings, critical review of the manuscript and
204 its dissemination.

205

206 **Results**

207 *Study sample*

208 Of the 9,551 patients with MI and complete data on sex, 25.1% (2,397) were women.
209 Compared with men, women were older (mean age 67.1 [SD 12.0] years vs. 63.1 [SD 11.7]
210 years), more likely to have hypertension (51.6 vs. 42.7%), COPD/asthma (16.3 vs. 11.7%),
211 and to present with NSTEMI (62.9 vs. 57.9%) (Table 1). Conversely, men were more
212 frequently smokers (68.9 vs. 62.2%), had higher rates of previous MI (17.6 vs. 14.3%),
213 previous PCI (10.5 vs. 8.1%), or previous coronary artery bypass graft (CABG) surgery (7.9
214 vs. 4.7%), and were more likely to undergo coronary intervention during the hospital stay
215 (48.6 vs. 41.6%) compared with women.

216

217 **Table 1** Patient baseline characteristics, stratified by sex

Variables	Men n=7,154	Women n=2,397	P value	Missing[¥]
NSTEMI, n. (%)	4,141 (57.9)	1,507 (62.9)	<0.001	0
Age, mean (SD), yr.	63.1 (11.7)	67.1 (12.0)	<0.001	19 (0.2)
White ethnicity, n. (%)	6,027 (96.9)	2,099 (98.5)	<0.001	1,197 (12.5)
IMD, median (IQR)	17.9 (10.7-31.4)	20.5 (11.8-33.4)	<0.001	5,258 (55.1)
BMI, mean(SD), kg/ m^2	28.6 (6.1)	28.9 (6.0)	0.151	3,366 (35.2)
Previous angina, n. (%)	1,339 (19.7)	451 (19.9)	0.826	493 (5.2)
Diabetes, n. (%)	1,256 (18.2)	457 (19.6)	0.133	329 (3.4)
Hypertension, n. (%)	2,904 (42.7)	1,170 (51.6)	<0.001	487 (5.1)

For peer review only - http://bmjopen.bmj.com/site/about/guidelines.xhtml

Variables	Men n=7,154	Women n=2,397	P value	Missing¥
Heart failure, n. (%)	150 (2.2)	62 (2.7)	0.151	503 (5.3)
Peripheral vascular disease, n. (%)	238 (3.6)	79 (3.6)	0.992	626 (6.6)
Cerebrovascular disease, n. (%)	305 (4.5)	123 (5.4)	0.065	496 (5.2)
Chronical renal failure, n. (%)	203 (3.0)	86 (3.8)	0.057	499 (5.2)
COPD/ asthma, n. (%)	796 (11.7)	370 (16.3)	<0.001	429 (4.5)
Smoker and ex-smoker, n. (%)	4,786 (68.9)	1,456 (62.2)	<0.001	263 (2.8)
CABG surgery, n. (%)	534 (7.9)	107 (4.7)	<0.001	496 (5.2)
Previous PCI, n. (%)	713 (10.5)	184 (8.1)	<0.001	510 (5.3)
Previous MI, n. (%)	1,196 (17.6)	324 (14.3)	0.0003	486 (5.1)
Cardiac rehabilitation† (n=9,307), n. (%)	6,387 (97.7)	2,110 (97.5)	0.565	607 (6.4)
Coronary intervention† (n=8,859) (PCI/CABG), n. (%)	2,810 (48.6)	826 (41.6)	<0.001	1,094 (12.4)
**Discharge medications†**				
Beta-blocker (n=8,029), n. (%)	5,691 (98.4)	1,888 (98.0)	0.166	322 (4.0)
ACE or ARB inhibitor (n=8,134), n. (%)	5,727 (97.8)	1,871 (97.0)	0.051	348 (4.3)
Statin (n=8,520), n. (%)	6,118 (99.1)	2,009 (98.9)	0.265	317 (3.7)
Aspirin (n=8,499), n. (%)	6,107 (99.4)	2,026 (99.0)	0.048	308 (3.6)
$P2Y_{12}$ inhibitors (n=5,491), n. (%)	3,610 (97.6)	1,259 (96.5)	0.037	486 (8.9)

Abbreviation: ACEi – angiotensin-converting enzyme inhibitor; ACS – Acute coronary syndrome; ARBs – Angiotensin receptor blocker, IMD indicates Index of Multiple Deprivation; CABG, coronary artery bypass graft; PCI, percutaneous coronary intervention; MI, Myocardial Infarction; COPD, chronic obstructive pulmonary disease; BMI, body mass index; NSTEMI, non ST-elevation myocardial infarction.¥15 (0.2%) patients had missing sex data. † Only patients eligible to receive treatments were included in the denominator of the complete cases.

For peer review only

Patterns of HRQoL

At baseline women had lower HRQoL compared with men: EQ VAS mean (SD) 59.8 (20.4) and vs. 64.5 (20.9), EQ VAS median (IQR) 60.00 (50.00 to 75.00) vs. 70.00 (50.00 to 80.00) and EQ-5D mean (SD) 0.66 (0.31) and vs. 0.74 (0.28), EQ-5D median (IQR) 0.73 (0.52 to 0.85) vs. 0.81 (0.62 to 1.00). The observed difference persisted through all-time points of follow-up (Figure 1). Over time HRQoL improved for both men and women following MI (Figure 1). Compared with men, women were more likely to report problems in all dimensions of EQ-5D (Figure 2). In the first month for both men and women, there was an increase in the proportion of patients reporting problems with usual activities, pain/discomfort and anxiety/depression (Figure 2). However, improvements were observed in the following months, with proportions of patients reporting pain/discomfort remaining stagnant (Figure 2).

Adjusted sex differences in HRQoL

The standardised differences showed that the weighting using the propensity scores balanced the systematic differences in baseline characteristics between men and women as the standardised differences were close or equal to zero (See Supplementary Table 2, which gives detail of the standardised differences). The minimum propensity score for each level was sufficiently greater than zero and that the maximum propensity score for each level was sufficiently less than 1, showing that the overlap assumption was not violated (Supplementary Figure 1). Compared with men, women had on average a lower HRQoL (adjusted EQ VAS model sex coefficient: -4.41, 95% CI -5.16 to -3.66 and adjusted EQ-5D model sex coefficient: -0.07, -0.08 to -0.06) and higher odds of reporting problems across all individual EQ-5D dimensions (Table 2). The interaction term exploring sex-based differences in the rates of HRQoL changes was not significant.

For peer review only

For peer review only - http://bmjopen.bmj.com/site/about/guidelines.xhtml

Table 2 Propensity score analysis to show health-related quality of life differences between women vs. men

**Health related quality of life**	**Coefficient (95% CI)**	**P value**
EQ VAS model		
Sex (Women vs. men)	-4.41 (-5.16 to -3.66)	<0.001
EQ 5D model		
Sex (Women vs. men)	-0.07 (-0.08 to -0.06)	<0.001
**EQ 5D dimensions**	**Odds ratio (95% CI)**	
Mobility problems model		
Sex (Women vs. men)	1.82 (1.58 to 2.09)	<0.001
Activities of daily living problems model		
Sex (Women vs. men)	1.70 (1.52 to 1.89)	<0.001
Self-care problems model		
Sex (Women vs. men)	1.75 (1.47 to 2.08)	<0.001
Pain/ discomfort model		
Sex (Women vs. men)	1.59 (1.45 to 1.75)	<0.001
Anxiety/ depression model		
Sex (Women vs. men)	2.03 (1.80 to 2.29)	<0.001

Factors associated with sex differences in HRQoL

Sex differences were observed in HRQoL in the bivariate model (EQ VAS model sex coefficient: -3.78, 95% CI -4.65 to -2.91 and EQ-5D model sex coefficient: -0.07, -0.08 to -0.06) (Table 3). The sex effect was markedly attenuated after accounting for patients' baseline HRQoL scores (EQ VAS coefficient: -2.56, 95% CI -3.38 to -1.73) (Table 3). However, for EQ-5D baseline scores did not attenuate the sex effect observed.

Table 3 Factors explaining sex differences observed in health related quality of life following MI

**Parameter**	**EQ-5D model, coefficient (95% CI)**	**EQ VAS model, coefficient (95% CI)**

For peer review only - http://bmjopen.bmj.com/site/about/guidelines.xhtml

Sex effect	-0.07 (-0.08 to -0.06)	-3.78 (-4.65 to -2.91)
Adding age, BMI, IMD	-0.06 (-0.07 to -0.05)	-3.33 (-4.20 to -2.40)
Adding pharmacotherapy and coronary intervention	-0.06 (-0.08 to -0.05)	-3.27 (-4.16 to -2.27)
Adding final diagnosis	-0.06 (-0.08 to -0.05)	-3.17 (-4.06 to -2.27)
Adding comorbidities and risk factors[¥]	-0.07 (-0.08 to -0.06)	-3.77 (-4.64 to -2.90)
Adding baseline value of the HRQoL metric*	-0.06 (-0.07 to -0.05)	-2.56 (-3.38 to -1.73)

Abbreviations: * Baseline EQ-5D for EQ-5D model and baseline EQVAS for EQ VAS model. BMI, body mass index; IMD indicates Index of Multiple Deprivation; MI, Myocardial Infarction; HRQoL, health related quality of life. [¥] Previous MI, age, previous coronary artery bypass graft surgery, smoking status, previous percutaneous coronary intervention, family history of coronary heart disease , peripheral vascular disease, hypercholesterolemia, previous angina, chronic obstructive pulmonary disease or asthma, diabetes mellitus, chronic renal failure, hypertension, cerebrovascular disease and heart failure.

For peer review only

Discussion

In this national longitudinal cohort study of 9,551 consecutive patients hospitalised with MI, we demonstrated that 1) women had lower HRQoL compared with men at baseline and throughout the following 12 months; 2) trajectories in HRQoL scores and all EQ-5D-3L domains (mobility, personal care, activities of daily living, pain/discomfort and anxiety/depression) assessed at 4 time points were similar between groups; and 3) adjustment for other variables, including age, risk factors, comorbidity, treatment, final diagnosis and baseline HRQoL decreased but did not eliminate the differences observed in HRQoL in women and men following MI.

To our knowledge, we present the largest longitudinal study to assess sex differences in MI survivors. Prior studies have addressed this question, however, they have been limited to small sample sizes[6-10], sub-selecting only MI patients receiving certain interventions [11], and short follow-up [11 22]. Data on 12-months HRQoL trajectories from contemporary real-world patient populations is limited. Previous research has shown that women report lower HRQoL at time of their presentation with MI[13 23], but the gender differences in baseline health status prior to MI have been attributed to the fact that women usually report more mental health disorders such as depression, fatigue or anxiety compared with men [24-26]. Our study, similarly to a recent large study of contemporary ACS patients treated with PCI, found that female sex was independently associated with significant impairment in all EQ-5D-3L domains (mobility, personal care, activities of daily living, pain/discomfort and anxiety/depression) [11]. Moreover, during longitudinal 12-months assessment women consistently reported lower HRQoL as measured by overall EQ-5D score, EQ VAS and problems at each of EQ-5D domains.

For peer review only - http://bmjopen.bmj.com/site/about/guidelines.xhtml

Between sex differences in epidemiology, pathophysiology, risk factors, clinical presentation and treatment strategies which have been demonstrated for MI patients are likely contributing to the observed differences in HRQoL following MI. In recent years, an increasing emphasis has been placed on the association between multi-morbidity level and negative outcomes in MI survivors. Indeed, the changes in HRQoL in men have been found to be associated with presenting characteristics of MI and complications of treatment while those of women were linked with their demographic characteristics and comorbidities[13 27]. Another study though suggested an impact of sex on physical functioning only, while gender-related factors such as femininity score, social support, and housework responsibility are independent predictors of long-term HRQoL[28]. Importantly however, after adjustment for multiple confounders, including comorbidities and treatment strategies such as medication, revascularization and cardiac rehabilitation, between sex differences in HRQoL remained significant for our study. Recognition of the associations of sex and gender itself with a diverse spectrum of factors related to cardiovascular and general health has led to a recently proposed concept of sex- and gender-sensitive medicine. Our study further magnifies this concept by demonstrating the lack of trend towards closing the gap during 12 months follow-up after MI in the large clinical practice-based patient population data from the EMMACE studies. In order to reduce the existing disparities, attempts should be sought to improve women's health status by identification of as many potential reasons as possible, addressing the modifiable risk factors and engaging more women into the recommended multidisciplinary post-MI management programmes.

In our study the meaningful improvement in HRQoL has been demonstrated equally in both men and women. Although our study is in line with a previous analysis of young MI populations in terms of general positive HRQoL trend after MI independently of sex [29], in

For peer review only - http://bmjopen.bmj.com/site/about/guidelines.xhtml

339 other studies different patterns have been found for women mainly reporting an improvement
340 in mental functioning while men tend to report improvement in the physical health status [8
341 30]. Analysis of magnitude of change in health status showed the highest increment
342 improvements of EQ-5D and EQ VAS score at 1 month after discharge and it's plateauing
343 after 6 months. Counterintuitively however, at 1 month time point the highest proportions of
344 patients reported some problems in anxiety/depression, pain/discomfort and usual activities.
345 This highlights that measuring not only health status via serial general HRQoL assessments
346 but also its domains are needed. Moreover, considering independent relationship between
347 EQ-5D and its domains in MI survivors and mortality[1], this strategy might provide
348 advantages in identification of patients at the highest risk of negative outcomes. Indeed,
349 analysis of 26,641 patients with first MI from SWEDEHEART registry have showed that
350 anxiety/depression assessed 6-10 weeks after MI is associated with 29% higher risk of
351 cardiovascular mortality and 34% higher risk of non-cardiovascular mortality independently
352 of traditional risk factors. Though these associations remained significant only if the mental
353 problems persisted after 12 months[31]. Another multinational study of HRQoL as assessed
354 by EQ-5D in 8978 post-MI patients showed that the presence of problems on 'self-care' and '
355 mobility' were most powerful predictors of all-cause mortality, whereas problems with
356 pain/discomfort and usual activities were most strongly associated with cardiovascular
357 events[1]. Future studies of sex-differences and targeted interventions after MI might help to
358 further personalize management strategies and as a result improve HRQoL outcomes in
359 women.

360

361 **Implications of the study**

362 Our findings build on other previous studies suggesting lower HRQoL in women compared
363 to men, but strengthen them by reporting absence of significant difference in patterns of

For peer review only - http://bmjopen.bmj.com/site/about/guidelines.xhtml

changes throughout 12 months follow-up in a broad real-world MI population. Higher adoption of serial assessment of patient-reported outcomes such as HRQoL are needed to tailor treatment interventions. The quantification of HRQoL at time of MI and identification of predictors of recovery may be important for designing targeted interventions tailored to meet the needs of patients and improve their physical and mental wellbeing.

Strengths and limitations

Our study has strengths in that it evaluates changes in HRQoL using nationwide longitudinal data which minimises selection bias and increases generalisability. There are no other databases of comparable size, coverage and quality. An inverse weighted propensity scoring approach was applied to weight data and balance out systematic differences based on observed covariates to minimise inherent bias. However, our study has limitations. 1) We used a generic quality of life metric rather than a disease-specific one to measure HRQoL. Nonetheless, the EQ-5D does capture dimensions of the quality of life that are relevant to, and are impacted by, MI such as mobility, depression/anxiety and pain/discomfort. In addition, EQ-5D has been validated in MI patients, and using a generic metric allows the comparison for the magnitude of HRQoL impairment between MI and other diseases. 2) The generalisability of the study's findings may be limited by a selection bias inherent as a result of loss to follow-up data. However, sensitivity analyses comparing those lost follow-up with those who were not showed minimal systematic differences (See Supplementary Table 3, which compares baseline characteristics of patients with complete follow-up with those missing one or more follow-up data points).

Conclusion

For peer review only - http://bmjopen.bmj.com/site/about/guidelines.xhtml

In this national longitudinal study, HRQoL improved for both men and women after MI. However, sex differences in HRQoL persisted over 12 months following MI, with women having a lower baseline and subsequent values of index EQ-5D score compared with men and higher levels of impairment in their mobility, self-care, usual activities, pain/discomfort and anxiety/ depression. Sex differences were attenuated by baseline HRQoL scores. Targeted interventions to address the reasons behind poor baseline health status could improve HRQoL recovery for MI survivors, especially for women.

Acknowledgements

We gratefully acknowledge the contributions from all hospitals, healthcare professionals and patients who participated in the EMMACE study. We would also like to thank Richard Gillott (IT support), Claire Forrest (Cardiology Research Coordinator) and Vera Hall (Finance support).

For peer review only - http://bmjopen.bmj.com/site/about/guidelines.xhtml

Contributorship statement

TBD analysed the data and drafted the manuscript. CPG and ASH contributed to the conception of the research, funding acquisition, project administration, supervision, study design and data collection. CPG, ASH, SA, AS, and BH provided expert clinical opinion and interpretation of the data. RMW, MH, TM and TBD provided statistical expert advice and interpretation of the data. GO was involved as a patient advisor in the interpretation of the research and the writing of the manuscript. All authors made critical revisions and provided intellectual content to the manuscript, approved the final version to be published and agreed to be accountable for all aspects of the work.

Competing interests

CPG reports personal fees from AstraZeneca, personal fees from Bayer, personal fees from Boehringer Inglehiem, personal fees from Amgen, personal fees from Daiichi Sankyo, personal fees from Vifor Pharma, grants from Abbott, grants from BMS, outside the submitted work. BH reports grants from National Institute for Health Research (NIHR/CS/009/004) and British Heart foundation (PG/19/54/34511), during the conduct of the study. AH reports personal fees (speaker honorarium) from NOVARTIS & SERVIER. AS acknowledges funding received from the European Society of Cardiology in form of an ESC Research Grant. The remaining authors have nothing to disclose. All authors have completed the ICMJE uniform disclosure form at www.icmje.org/coi_disclosure.pdf.

Funding

This work was supported by the National Institute for Health Research (NIHR/CS/009/004), and British Heart Foundation (PG/19/54/34511). MH was funded by the Wellcome Trust (grant reference: reference: 206470/Z/17/Z).

For peer review only

For peer review only - http://bmjopen.bmj.com/site/about/guidelines.xhtml

438 **Data sharing statement**

439 The datasets used and/or analysed during the current study are available from the principal
440 investigator of the study on reasonable request.

441

442 **Ethics**

443 EMMACE-3 has been given a favourable ethical opinion by the Leeds (Central) Research
444 Ethics committee (REC reference: 10/H1313/74), is registered on ClinicalTrials.gov
445 (NCT01808027) and has been adopted onto the National Institute for Health Research
446 Comprehensive Research Network portfolio (9102). EMMACE-4 has been given favourable
447 ethical opinion by the West Midlands - Black Country Research Ethics Committee (REC
448 reference: 12/WM/0431), is registered on ClinicalTrials.gov (NCT01819103) and has been
449 adopted onto the National Institute for Health Research Comprehensive Research Network
450 portfolio (9102)

451

452 **Word count:** Abstract:	295
453 Main text excluding tables, figures and references:	2,834
454 Tables:	3
455 Figures:	2

456
457
458
459
460

For peer review only - http://bmjopen.bmj.com/site/about/guidelines.xhtml

References

1. Pocock S, Brieger DB, Owen R, et al. Health-related quality of life 1–3 years post-myocardial infarction: its impact on prognosis. Open Heart 2021;**8**(1):e001499

2. Dreyer RP, Ranasinghe I, Wang Y, et al. Sex differences in the rate, timing, and principal diagnoses of 30-day readmissions in younger patients with acute myocardial infarction. Circulation 2015;**132**(3):158-66

3. Dreyer RP, Dharmarajan K, Kennedy KF, et al. Sex differences in 1-year all-cause rehospitalization in patients after acute myocardial infarction: a prospective observational study. Circulation 2017;**135**(6):521-31

4. Vaccarino V, Rathore SS, Wenger NK, et al. Sex and racial differences in the management of acute myocardial infarction, 1994 through 2002. N Engl J Med 2005;**353**(7):671-82

5. Canto JG, Rogers WJ, Goldberg RJ, et al. Association of age and sex with myocardial infarction symptom presentation and in-hospital mortality. JAMA 2012;**307**(8):813-22

6. Emery CF, Frid DJ, Engebretson TO, et al. Gender differences in quality of life among cardiac patients. Psychosom. Med. 2004;**66**(2):190-97

7. Beck CA, Joseph L, Bélisle P, Pilote L, Investigators Q. Predictors of quality of life 6 months and 1 year after acute myocardial infarction. Am Heart J 2001;**142**(2):271-79

8. Brink E, Grankvist G, Karlson BW, Hallberg LR-M. Health-related quality of life in women and men one year after acute myocardial infarction. Qual Life Res 2005;**14**(3):749-57

9. Lacey E, Walters S. Continuing inequality: gender and social class influences on self perceived health after a heart attack. J. Epidemiol. Community Health 2003;**57**(8):622-27

10. Worcester MUC, Murphy BM, Elliott PC, et al. Trajectories of recovery of quality of life in women after an acute cardiac event. Br J Health Psychol 2007;**12**(1):1-15

11. Koh Y, Stehli J, Martin C, et al. Does sex predict quality of life after acute coronary syndromes: an Australian, state-wide, multicentre prospective cohort study. BMJ open 2019;**9**(12)

12. Dreyer RP, Wang Y, Strait KM, et al. Gender differences in the trajectory of recovery in health status among young patients with acute myocardial infarction: results from the variation in recovery: role of gender on outcomes of young AMI patients (VIRGO) study. Circulation 2015;**131**(22):1971-80

13. Dueñas M, Ramirez C, Arana R, Failde I. Gender differences and determinants of health related quality of life in coronary patients: a follow-up study. BMC Cardiovasc. Disord. 2011;**11**(1):24

14. Alabas O, West R, Gillott R, et al. Evaluation of the Methods and Management of Acute Coronary Events (EMMACE)-3: protocol for a longitudinal study. BMJ open 2015;**5**(6):e006256

For peer review only - http://bmjopen.bmj.com/site/about/guidelines.xhtml

15. Herrett E, Smeeth L, Walker L, Weston C, Group MA. The Myocardial Ischaemia National Audit Project (MINAP). Heart 2010;**96**(16):1264-7 doi: 10.1136/hrt.2009.192328[published Online First: Epub Date]|.

16. Cheung K, Oemar M, Oppe M, Rabin R. EQ-5D user guide: basic information on how to use EQ-5D. Rotterdam: EuroQol Group 2009

17. Dolan P. Modeling valuations for EuroQol health states. Med care

1997:1095-108

18. Nowels D, McGloin J, Westfall JM, Holcomb S. Validation of the EQ-5D quality of life instrument in patients after myocardial infarction. Qual Life Res 2005;**14**(1):95-105

19. Nolan CM, Longworth L, Lord J, et al. The EQ-5D-5L health status questionnaire in COPD: validity, responsiveness and minimum important difference. Thorax 2016;**71**(6):493-500

20. Cattle B, Baxter P, Greenwood D, Gale C, West R. Multiple imputation for completion of a national clinical audit dataset. Stat Med 2011;**30**(22):2736-53

21. Rubin DB. *Multiple imputation for nonresponse in surveys*: John Wiley & Sons, 2004.

22. Failde II, Soto MM. Changes in health related quality of life 3 months after an acute coronary syndrome. BMC Public Health 2006;**6**(1):18

23. Norris CM, Spertus JA, Jensen L, Johnson J, Hegadoren KM, Ghali WA. Sex and gender discrepancies in health-related quality of life outcomes among patients with established coronary artery disease. Circ Cardiovasc Qual Outcomes 2008;**1**(2):123-30

24. Dreyer RP, Smolderen KG, Strait KM, et al. Gender differences in pre-event health status of young patients with acute myocardial infarction: A VIRGO study analysis. Eur. Heart J. Acute Cardiovasc. Care 2016;**5**(1):43-54

25. Barnett K, Mercer SW, Norbury M, Watt G, Wyke S, Guthrie B. Epidemiology of multimorbidity and implications for health care, research, and medical education: a cross-sectional study. Lancet 2012;**380**(9836):37-43

26. Parashar S, Rumsfeld JS, Reid KJ, et al. Impact of depression on sex differences in outcome after myocardial infarction. Circ Cardiovasc Qual Outcomes 2009;**2**(1):33-40

27. Höfer S, Benzer W, Oldridge N. Change in health-related quality of life in patients with coronary artery disease predicts 4-year mortality. Int. J. Cardiol. 2014;**174**(1):7-12

28. Leung Yinko SS, Pelletier R, Behlouli H, et al. Health-Related quality of life in premature acute coronary syndrome: does patient sex or gender really matter? J. Am. Heart Assoc. 2014;**3**(4):e000901

29. Dreyer RP, Zheng X, Xu X, et al. Sex differences in health outcomes at one year following acute myocardial infarction: a report from the China Patient-Centered Evaluative Assessment of Cardiac Events prospective acute myocardial infarction study. Eur. Heart J. Acute Cardiovasc. Care 2019;**8**(3):273-82

For peer review only

For peer review only - http://bmjopen.bmj.com/site/about/guidelines.xhtml

30. Staniūtė M, Brožaitienė J. Changes in health-related quality of life among patients with coronary artery disease: a 2-year follow-up. Medicina 2010;**46**(12):843

31. Lissåker CT, Norlund F, Wallert J, Held C, Olsson EM. Persistent emotional distress after a first-time myocardial infarction and its association to late cardiovascular and non-cardiovascular mortality. Eur. J. Prev. Cardiol. 2019;**26**(14):1510-18

For peer review only

Figure Legends

Figure 1 Health related quality of life trajectories following myocardial infarction by sex and UK general population

Figure 2 Health related quality of life domains trajectories following myocardial infarction by sex

For peer review only

For peer review only - http://bmjopen.bmj.com/site/about/guidelines.xhtml

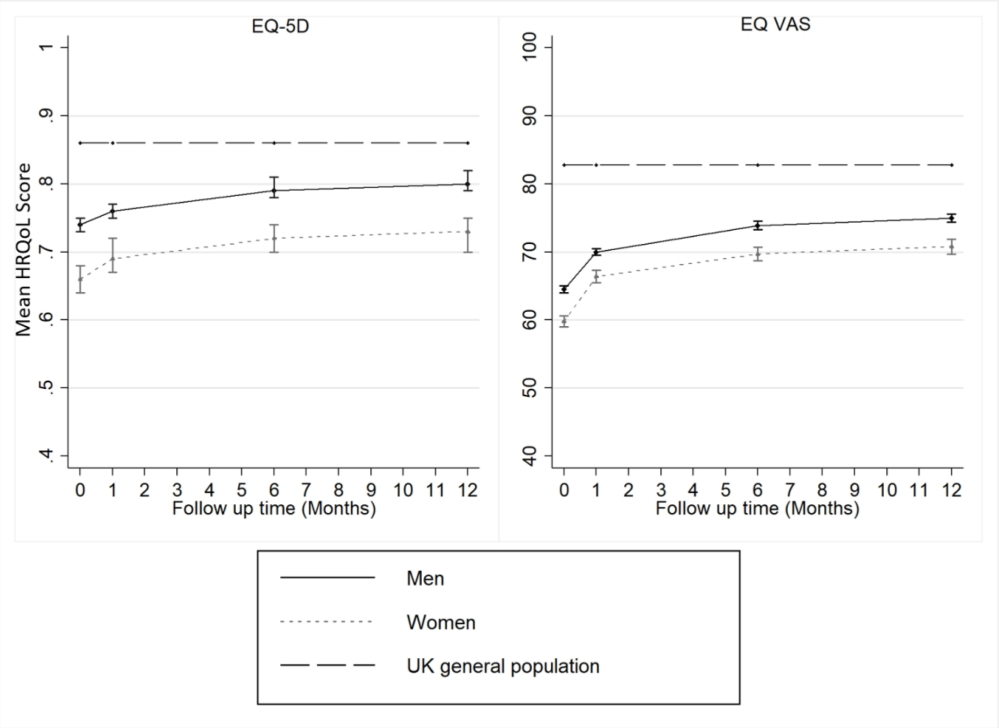

Figure 1

254x184mm (150 x 150 DPI)

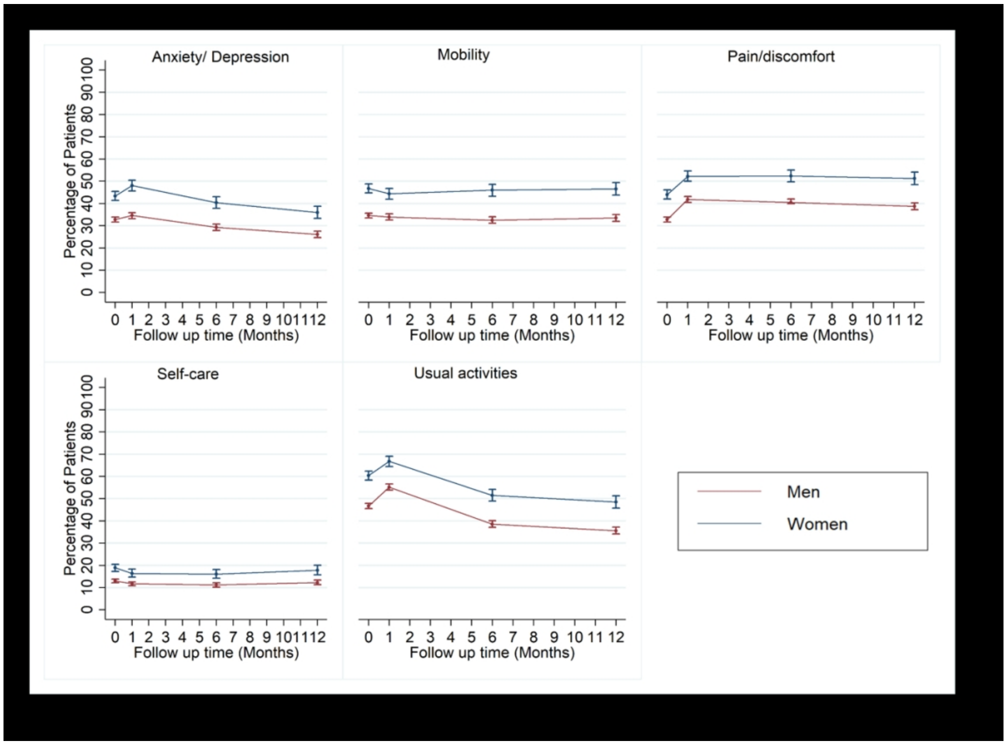

Figure 2

227x167mm (150 x 150 DPI)

SUPPLEMENTARY MATERIAL

Title: Sex differences in health-related quality of life trajectories following acute myocardial infarction: National longitudinal cohort study.

Authors

T.B. Dondo (PhD), T. Munyombwe (PhD), M. Hall (PhD), B. Hurdus (MBBS), A.Soloveva (PhD)[1,2], G. Oliver (Patient), S. Aktaa (MD), RM West (DPhil), A.S. Hall (FRCP), C.P. Gale (PhD FRCP).

For peer review only

Section 1: Supplementary methods

Propensity score analysis

A non-parsimonious multivariable logistic regression model was used to derive propensity scores (PS) (probability of being a man or a woman, conditional on observed patient baseline covariates) to weight the data balancing out systematic differences. The model was adjusted for patient baseline characteristics; AMI phenotype (STEMI vs. NSTEMI), age, body mass index (BMI), index of multiple deprivation (IMD) score, smoking status (never vs. current or ex-smoker), family history of coronary heart disease (CHD), previous angina, history of diabetes mellitus, hypertension, heart failure, peripheral vascular disease, cerebrovascular disease, chronic renal failure, chronic obstructive pulmonary disease (COPD) or asthma, hypercholesterolemia, previous percutaneous coronary intervention (PCI), previous coronary artery bypass graft (CABG) surgery, previous AMI, coronary intervention, hospital discharge medications (aspirin, β blockers, statins, ACEi/ARB and $P2Y_{12}$ inhibitors) and referral for cardiac rehabilitation. In order to assess whether the weights constructed from the propensity score model balanced the covariates between men and women, standardised differences were derived, a perfectly balanced covariate has a standardised difference of zero. Violation of the overlap assumption was assessed using an overlap plot and by summarising the estimated probabilities of sex. Supplemental Figure 1 illustrates the results of the assessment of the overlap assumption and shows that the minimum propensity score for each level was sufficiently greater than zero and that the maximum propensity score for each level was sufficiently less than 1, thus the assumption was not violated. The area under the curve for the propensity score model was 0.64, which indicated moderate discrimination for the model.

For peer review only

Supplementary Figure 1. Overlap assumption assessment plots (A) and Distribution of propensity scores across comparison groups (B).

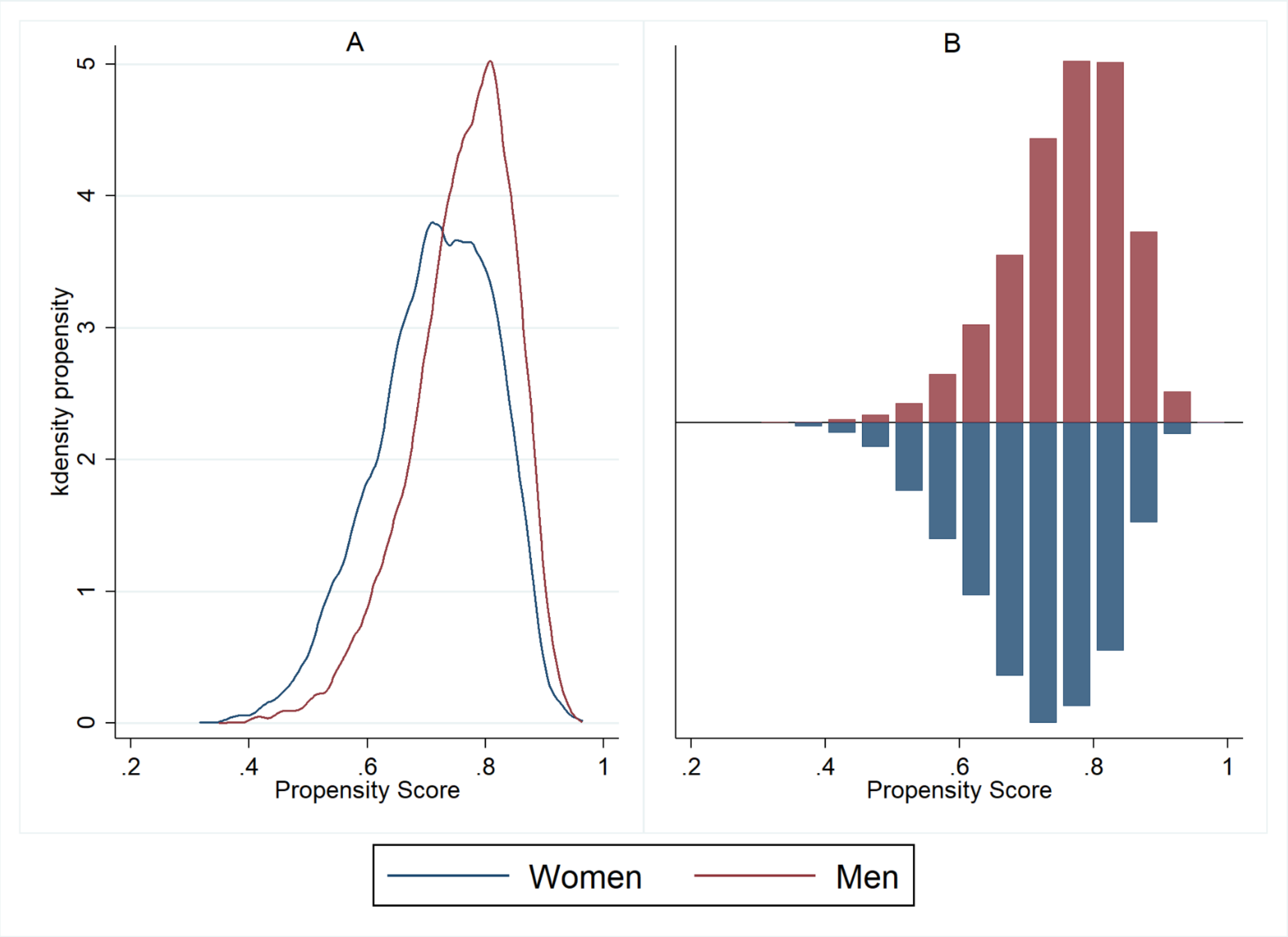

Section 2: Supplementary methods

Handling missing data in baseline characteristics

Missing data were imputed using multiple imputations by chained equations. Ten imputed datasets were derived from 20 iterations. Data were imputed for missing baseline characteristics and not follow-up outcome data. A default imputation (missing data default imputed to "NO") strategy based on clinical expert opinion was implemented for cardiovascular history, cardiovascular risk factors, and categorical treatment variables. The imputation strategy applied is summarised in Supplemental Table 1.

Supplementary Table 1. Imputation Strategy

Variable	Variable Type	Missing n (%)	Imputation Method
Sex	Binary	15 (0.16)	Predictor/ Auxiliary
Age	Continuous	19 (0.20)	Predictive mean matching
Family history of CHD	Binary	1,473 (15.40)	Predictor/ Auxiliary and Default imputed
Previous PCI	Binary	510 (5.33)	Predictor/ Auxiliary and Default imputed
Previous CABG	Binary	496 (5.19)	Predictor/ Auxiliary and Default imputed
Overallimdscore	Continuous	5,260 (55.00)	Predictive mean matching
BMI	Continuous	3,374 (35.27)	Predictive mean matching
Cardiac rehabilitation referral at hospital discharge	Binary	609 (6.37)	Predictor/ Auxiliary and Default imputed
Previous AMI	Binary	486 (5.08)	Predictor/ Auxiliary and Default imputed
Previous Angina	Binary	493 (5.15)	Predictor/ Auxiliary and Default imputed
Hypertension	Binary	487 (5.09)	Predictor/ Auxiliary and Default imputed
Hypercholesterolaemia	Binary	567 (5.93)	Predictor/ Auxiliary and Default imputed

For peer review only

Variable	Variable Type	Missing n (%)	Imputation Method
Peripheral Vascular Disease	Binary	626 (6.54)	Predictor/ Auxiliary and Default imputed
Cerebrovascular Disease	Binary	496 (5.19)	Predictor/ Auxiliary and Default imputed
Asthma or COPD	Binary	506 (5.29)	Predictor/ Auxiliary and Default imputed
Chronic Renal Failure	Binary	499 (5.22)	Predictor/ Auxiliary and Default imputed
Diabetes	Binary	330 (3.45)	Predictor/ Auxiliary and Default imputed
Reinfarction	Binary	510 (5.33)	Predictor/ Auxiliary and Default imputed
EQ-5D at admission	Continuous	366 (3.83)	Predictor/ Auxiliary
EQVAS at admission	Continuous	303 (3.17)	Predictor/ Auxiliary
Mobility	Binary	234 (2.45)	Predictor/ Auxiliary
Self-care	Binary	250 (2.61)	Predictor/ Auxiliary
Usual activities	Binary	300 (3.14)	Predictor/ Auxiliary
Pain/discomfort	Binary	250 (2.61)	Predictor/ Auxiliary
Anxiety/depression	Binary	252 (2.63)	Predictor/ Auxiliary

Abbreviations: CABG, coronary artery bypass graft; PCI, percutaneous coronary intervention; MI, Myocardial Infarction; COPD, chronic obstructive pulmonary disease; BMI, body mass index; CHD, coronary heart disease.

For peer review only

Section 3: Supplementary Tables

The balance check results are summarised in Supplemental Table 2, which shows that the standardised differences for variables in the weighted data were close to zero. The diagnostic assessments suggest that weighting by the inverse probability of sex created a sample in which the distributions of the covariates were similar between men and women.

Supplementary Table 2. Covariate balance across men and women after weighting on the propensity score

Variable	Mean in men	Mean in women	Standardized difference
Age	62.28	65.62	-0.285
Deprivation (IMD score)	22.55	24.68	-0.135
BMI	28.46	29.05	-0.108
Smoker ever	0.69	0.64	0.118
Final diagnosis	0.44	0.41	0.058
Family history of CHD	0.40	0.40	0.003
Previous PCI	0.07	0.06	0.023
Previous CABG	0.06	0.04	0.092
Previous MI	0.13	0.09	0.132
Hypertension	0.43	0.53	-0.201
Hypercholesterolaemia	0.34	0.34	-0.008
PVD	0.03	0.04	-0.049
CVSD	0.04	0.04	0.038
COPD	0.11	0.16	-0.126
Chronic renal failure	0.02	0.04	-0.106
Chronic cardiac failure	0.01	0.01	0.033
Diabetes	0.15	0.17	-0.046
Discharge medications			
Aspirin	0.89	0.87	0.050
β blockers	0.83	0.83	-0.002
Statin	0.89	0.88	0.036
ACEi/ARBs	0.85	0.83	0.056
$P2Y_{12}$ inhibitors	0.78	0.77	0.015

For peer review only

Coronary intervention	0.52	0.45	0.139
Cardiac rehabilitation	0.93	0.93	0.005

Abbreviations: ACEi – angiotensin-converting enzyme inhibitor; ACS – Acute coronary syndrome; ARBs – Angiotensin receptor blocker, IMD indicates Index of Multiple Deprivation; CABG, coronary artery bypass graft; PCI, percutaneous coronary intervention; MI, Myocardial Infarction; COPD, chronic obstructive pulmonary disease; CVSD cerebrovascular disease; BMI, body mass index.

Supplementary Table 3. Patient baseline characteristics (patients with complete follow-up data vs patients with missing follow-up data at one or time points).

Variables	Patients with complete follow up data at all-time points (n= 3,413)	Patients with missing follow-up data at one or time points (n= 6,138)	P value
NSTEMI, n. (%)	2,051 (60.1)	3,597 (58.6)	0.155
Age, mean (SD), yr.	65.9 (10.6)	63.1 (12.5)	<0.001
White ethnicity, n. (%)	2,915 (98.7)	5,211 (96.5)	<0.001
IMD, median (IQR)	16.3 (10.2-27.1)	20.6 (11.9-34.0)	<0.001
BMI, mean(SD), kg/ m^2	27.4 (24.9-30.8)	28.1 (25.1-31.6)	<0.001
Previous angina, n. (%)	619 (19.0)	1,171 (20.2)	0.193
Diabetes, n. (%)	506 (15.4)	1,207 (20.3)	<0.001
Hypertension, n. (%)	1,486 (45.7)	2,588 (44.5)	0.303
Heart failure, n. (%)	62 (1.9)	150 (2.6)	0.042
Peripheral vascular disease, n. (%)	97 (3.1)	220 (3.8)	0.056
Cerebrovascular disease, n. (%)	136 (4.2)	292 (5.0)	0.069
Chronical renal failure, n. (%)	76 (2.3)	213 (3.7)	<0.001
COPD, n. (%)	374 (11.5)	792 (13.7)	0.004
Smoker and ex-smoker, n. (%)	2,050 (62.0)	4,192 (70.1)	<0.001
CABG surgery, n. (%)	227 (7.0)	414 (7.1)	0.826

For peer review only

Variables	Patients with complete follow up data at all-time points (n= 3,413)	Patients with missing follow-up data at one or time points (n= 6,138)	P value
Previous PCI, n. (%)	300 (9.3)	597 (10.3)	0.119
Previous AMI, n. (%)	485 (14.9)	1,035 (17.8)	<0.001
Cardiac rehabilitation† (n=8,700), n. (%)	3,049 (97.8)	5,448 (97.6)	0.681
Coronary intervention† (n=7,261), n. (%)	1,563 (60.3)	2,765 (59.2)	0.353
**Discharge medications†**			
Beta-blocker (n=7,708), n (%)	2,741 (98.4)	4,838 (98.3)	0.764
ACEi or ARB inhibitor (n=7,786), n. (%)	2,742 (97.8)	4,856 (97.5)	0.467
Statin (n=8,203), n. (%)	2,911 (99.3)	5,216 (99.0)	0.214
Aspirin (n=8,191), n. (%)	2,920 (99.3)	5,213 (99.3)	0.822
$P2Y_{12}$ inhibitors (n=5,005), n. (%)	1,828 (97.4)	3,041 (97.2)	0.593

Abbreviations: ACEi – angiotensin-converting enzyme inhibitor; ACS – Acute coronary syndrome; ARBs – Angiotensin receptor blocker, IMD indicates Index of Multiple Deprivation; CABG, coronary artery bypass graft; PCI, percutaneous coronary intervention; MI, Myocardial Infarction; COPD, chronic obstructive pulmonary disease; BMI, body mass index; NSTEMI, non ST-elevation myocardial infarction.

For peer review only

STROBE Statement—checklist of items that should be included in reports of observational studies

	Item No	Recommendation	Page number	
**Title and abstract**	1	(*a*) Indicate the study's design with a commonly used term in the title or the abstract	1	✓
		(*b*) Provide in the abstract an informative and balanced summary of what was done and what was found	2-3	✓
**Introduction**				
Background/rationale	2	Explain the scientific background and rationale for the investigation being reported	5-6	✓
Objectives	3	State specific objectives, including any prespecified hypotheses	5-6	✓
**Methods**				
Study design	4	Present key elements of study design early in the paper	6	✓
Setting	5	Describe the setting, locations, and relevant dates, including periods of recruitment, exposure, follow-up, and data collection	6	✓
Participants	6	(*a*) *Cohort study*—Give the eligibility criteria, and the sources and methods of selection of participants. Describe methods of follow-up<br>*Case-control study*—Give the eligibility criteria, and the sources and methods of case ascertainment and control selection. Give the rationale for the choice of cases and controls<br>*Cross-sectional study*—Give the eligibility criteria, and the sources and methods of selection of participants	6	✓
		(*b*) *Cohort study*—For matched studies, give matching criteria and number of exposed and unexposed<br>*Case-control study*—For matched studies, give matching criteria and the number of controls per case		NA
Variables	7	Clearly define all outcomes, exposures, predictors, potential confounders, and effect modifiers. Give diagnostic criteria, if applicable	6-8	✓
Data sources/ measurement	8*	For each variable of interest, give sources of data and details of methods of assessment (measurement). Describe comparability of assessment methods if there is more than one group	6-8	✓
Bias	9	Describe any efforts to address potential sources of bias	7-8	✓
Study size	10	Explain how the study size was arrived at	6	✓
Quantitative variables	11	Explain how quantitative variables were handled in the analyses. If applicable, describe which groupings were chosen and why	7	✓
Statistical methods	12	(*a*) Describe all statistical methods, including those used to control for confounding	7-8	✓
		(*b*) Describe any methods used to examine subgroups and interactions	8	✓
		(*c*) Explain how missing data were addressed	8	✓
		(*d*) *Cohort study*—If applicable, explain how loss to follow-up was addressed<br>*Case-control study*—If applicable, explain how matching of cases and controls was addressed		N/A

For peer review only

		Cross-sectional study—If applicable, describe analytical methods taking account of sampling strategy		
		(*e*) Describe any sensitivity analyses	N/A	

Continued on next page

For peer review only

For peer review only - http://bmjopen.bmj.com/site/about/guidelines.xhtml

**Results**				
Participants	13*	(a) Report numbers of individuals at each stage of study—eg numbers potentially eligible, examined for eligibility, confirmed eligible, included in the study, completing follow-up, and analysed	-	
		(b) Give reasons for non-participation at each stage	-	
		(c) Consider use of a flow diagram	-	
Descriptive data	14*	(a) Give characteristics of study participants (eg demographic, clinical, social) and information on exposures and potential confounders	9-10	✓
		(b) Indicate number of participants with missing data for each variable of interest	9-10	✓
		(c) *Cohort study*—Summarise follow-up time (eg, average and total amount)	11	✓
Outcome data	15*	*Cohort study*—Report numbers of outcome events or summary measures over time	11	✓
		Case-control study—Report numbers in each exposure category, or summary measures of exposure		
		Cross-sectional study—Report numbers of outcome events or summary measures		
Main results	16	(*a*) Give unadjusted estimates and, if applicable, confounder-adjusted estimates and their precision (eg, 95% confidence interval). Make clear which confounders were adjusted for and why they were included	11-13	✓
		(*b*) Report category boundaries when continuous variables were categorized		NA
		(*c*) If relevant, consider translating estimates of relative risk into absolute risk for a meaningful time period		NA
Other analyses	17	Report other analyses done—eg analyses of subgroups and interactions, and sensitivity analyses	11-13	✓
**Discussion**				
Key results	18	Summarise key results with reference to study objectives	14	✓
Limitations	19	Discuss limitations of the study, taking into account sources of potential bias or imprecision. Discuss both direction and magnitude of any potential bias	17	✓
Interpretation	20	Give a cautious overall interpretation of results considering objectives, limitations, multiplicity of analyses, results from similar studies, and other relevant evidence	14-18	✓
Generalisability	21	Discuss the generalisability (external validity) of the study results	17	✓
**Other information**				
Funding	22	Give the source of funding and the role of the funders for the present study and, if applicable, for the original study on which the present article is based	19	✓

*Give information separately for cases and controls in case-control studies and, if applicable, for exposed and unexposed groups in cohort and cross-sectional studies.

Note: An Explanation and Elaboration article discusses each checklist item and gives methodological background and published examples of transparent reporting. The STROBE checklist is best used in conjunction with this article (freely available on the Web sites of PLoS Medicine at http://www.plosmedicine.org/, Annals of Internal Medicine at http://www.annals.org/, and Epidemiology at http://www.epidem.com/). Information on the STROBE Initiative is available at www.strobe-statement.org.

For peer review only

BMJ Open

Sex differences in health-related quality of life trajectories following myocardial infarction: National longitudinal cohort study.

Journal:	*BMJ Open*
Manuscript ID	bmjopen-2022-062508.R1
Article Type:	Original research
Date Submitted by the Author:	19-Jul-2022
Complete List of Authors:	Dondo, Tatendashe; University of Leeds, Leeds Institute of Cardiovascular and Metabolic Medicine; University of Leeds, Leeds Institute for Data Analytics<br>Munyombwe, T; University of Leeds, Leeds Institute of Cardiovascular and Metabolic Medicine; University of Leeds, Leeds Institute for Data Analytics<br>Hall, Marlous ; University of Leeds, Leeds Institute of Cardiovascular and Metabolic Medicine ; University of Leeds, Leeds Institute for Data Analytics<br>Hurdus, B; University of Leeds, Leeds institute for data analytics; Leeds Teaching Hospitals NHS Trust, Department of Cardiology<br>Soloveva, A.; University of Leeds, Leeds Institute of Cardiovascular and Metabolic Medicine; University of Leeds, Leeds Institute for Data Analytics<br>Oliver, Gerard; West yorkshire<br>Aktaa, Suleman; University of Leeds, Leeds Institute of Cardiovascular and Metabolic Medicine ; Leeds General Infirmary, Department of Cardiology Leeds General Infirmary<br>West, Robert; University of Leeds, Leeds Institute of Health Sciences<br>Hall, Alistair; Leeds General Infirmary Department of Cardiology, Cardiology Department; University of Leeds, Leeds Institute of Cardiovascular and Metabolic Medicine<br>Gale, Chris; University of Leeds, Leeds Institute of Cardiovascular and Metabolic Medicine ; Leeds General Infirmary, Department of Cardiology Leeds General Infirmary
**Primary Subject Heading**:	Cardiovascular medicine
Secondary Subject Heading:	Cardiovascular medicine
Keywords:	Myocardial infarction < CARDIOLOGY, Epidemiology < TROPICAL MEDICINE, Coronary heart disease < CARDIOLOGY

For peer review only - http://bmjopen.bmj.com/site/about/guidelines.xhtml

I, the Submitting Author has the right to grant and does grant on behalf of all authors of the Work (as defined in the below author licence), an exclusive licence and/or a non-exclusive licence for contributions from authors who are: i) UK Crown employees; ii) where BMJ has agreed a CC-BY licence shall apply, and/or iii) in accordance with the terms applicable for US Federal Government officers or employees acting as part of their official duties; on a worldwide, perpetual, irrevocable, royalty-free basis to BMJ Publishing Group Ltd ("BMJ") its licensees and where the relevant Journal is co-owned by BMJ to the co-owners of the Journal, to publish the Work in this journal and any other BMJ products and to exploit all rights, as set out in our licence.

The Submitting Author accepts and understands that any supply made under these terms is made by BMJ to the Submitting Author unless you are acting as an employee on behalf of your employer or a postgraduate student of an affiliated institution which is paying any applicable article publishing charge ("APC") for Open Access articles. Where the Submitting Author wishes to make the Work available on an Open Access basis (and intends to pay the relevant APC), the terms of reuse of such Open Access shall be governed by a Creative Commons licence – details of these licences and which Creative Commons licence will apply to this Work are set out in our licence referred to above.

Other than as permitted in any relevant BMJ Author's Self Archiving Policies, I confirm this Work has not been accepted for publication elsewhere, is not being considered for publication elsewhere and does not duplicate material already published. I confirm all authors consent to publication of this Work and authorise the granting of this licence.

For peer review only

Sex differences in health-related quality of life trajectories following myocardial infarction: National longitudinal cohort study.

Authors

T.B. Dondo (PhD)[1,2], T. Munyombwe (PhD)[1,2], M. Hall (PhD)[1,2], B. Hurdus (MBBS)[1,2,3], A.Soloveva (PhD)[1,2], G. Oliver (Patient)[4], S. Aktaa (MD)[1,2,3] ,R.M. West (DPhil)[5], A.S. Hall (FRCP)[1,3], C.P. Gale (PhD FRCP)[1,2,3].

Affiliations

[1]Leeds Institute of Cardiovascular and Metabolic Medicine, University of Leeds, Leeds, UK

[2] Leeds Institute for Data Analytics, University of Leeds, Leeds, United Kingdom

[3] Department of Cardiology Leeds General Infirmary, Leeds, UK

[4]Patient representative, Lancashire, United Kingdom

[5]Leeds Institute of Health Sciences, University of Leeds, Leeds, United Kingdom

Correspondence

Dr Theresa Munyombwe

Leeds Institute of Cardiovascular and Metabolic Medicine,

MRC Bioinformatics Centre, Level 11, Worsley building, Clarendon way,

Leeds, LS2 9JT

Email: t.munyombwe@leeds.ac.uk

Tel: 0044 (0)113 343 0241

Twitter @munyomb1

For peer review only

For peer review only - http://bmjopen.bmj.com/site/about/guidelines.xhtml

Abstract

Objectives: To investigate sex-based differences in baseline values and longitudinal trajectories of health-related quality of life (HRQoL) in a large cohort of myocardial infarction (MI) survivors after adjusting for other important factors.

Design: Longitudinal cohort study

Setting: Population-based longitudinal study the Evaluation of the Methods and Management of Acute Coronary Events (EMMACE) study linked with national cardiovascular registry. Data was collected from 77 hospitals in England between 1 November 2011 and 24 June 2015.

Participants: 9,551 patients with MI. Patients were eligible for the study if they were ≥18 years of age.

Primary and secondary outcome measures: HRQoL was measured by EuroQol five-dimension (EQ-5D, EQ VAS) survey at baseline, 1, 6 and 12 months after discharge. Multi-level linear and logistic regression models coupled with inverse probability weighted propensity scoring were used to evaluate sex differences in HRQoL following MI.

Results: Of the 9,551 patients with MI and complete data on sex, 25.1% (2,397) were women. At baseline, women reported lower HRQoL (EQ VAS [mean (SD) 59.8 (20.4) vs. 64.5 (20.9)] [median (IQR) 60.00 (50.00 to 75.00) vs. 70.00 (50.00 to 80.00)]) (EQ-5D [mean (SD) 0.66 (0.31) vs. 0.74 (0.28)] [median (IQR) 0.73 (0.52 to 0.85) vs. 0.81 (0.62 to 1.00)]) and were more likely to report problems in each HRQoL domain compared with men. In the covariate balanced and adjusted multi-level models sex differences in HRQoL persisted during follow-up, with lower EQ VAS and EQ-5D scores in women compared with men (adjusted EQ VAS model sex coefficient: -4.41, 95% CI -5.16 to -3.66 and adjusted EQ-5D model sex coefficient: -0.07, 95% CI -0.08 to -0.06).

For peer review only

Conclusions: Women have lower HRQoL compared with men at baseline and during 12 months follow-up after MI. Tailored interventions for women following an MI could improve their quality of life.

Clinical Trial Registration: ClinicalTrials.gov (NCT04598048, NCT01808027, NCT01819103)

Keywords: Quality of life, Women, Myocardial infarction, Epidemiology, Acute coronary syndrome

For peer review only

For peer review only - http://bmjopen.bmj.com/site/about/guidelines.xhtml

Strengths and limitations of this study

- Data source is linked nationwide longitudinal HRQoL data which minimises selection bias and increases generalisability.
- An inverse weighted propensity scoring approach was applied to weight data and balance out systematic differences based on observed covariates to minimise inherent bias.
- Used generic quality of life metric rather than a disease-specific one to measure HRQoL following MI.
- Potential selection bias due to loss to follow-up.

For peer review only

Introduction

Recent decades were characterized by significant decline of mortality in myocardial infarction (MI). Consequently, health-related quality of life (HRQoL) following MI emerged as another important indicator of patient care. HRQoL represents patients' perspective of their health state but also serves as an important clinical risk marker and treatment target given lower HRQoL in MI survivors is independently associated with increased risk of death[1].

Emerging evidence points to the significant sex-based differences in MI population that may also account for HRQoL differences. The exact explanation for this phenomenon remains uncertain, but distinct clinical presentation and etiology of MI, higher age and comorbidity burden, less frequent invasive therapeutic approach, higher rehospitalisation rates and long-term mortality had been consistently shown in women compared with men [2-5]. Importantly, these differences in characteristics and treatment strategies may impact not only HRQoL at the time of the acute event but also its trajectories over time. Previous studies that demonstrated lower HRQoL scores in women compared with men were either small [6-10] or focused on a selected subgroup of MI patients [11 12] thus were unable to adjust for multiple confounding factors or answer the question of independent sex differences in a heterogeneous MI population. Moreover, only a few contemporary studies explored longitudinal HRQoL estimates depending on sex [6 8-10 12 13] thus an appropriate time for the subsequent assessment of HRQoL remains unknown. Knowledge of such sex-based disparities in HRQoL is important as it could highlight the need for strategies to improve the health status of women following MI. Furthermore, understanding the differences in the HRQoL domains may provide an opportunity to identify the components of patient-reported health that need particularly higher attention and clinical counseling. Using a large nationwide longitudinal

For peer review only - http://bmjopen.bmj.com/site/about/guidelines.xhtml

125 cohort study of consecutive patients hospitalised with acute coronary syndrome (ACS) we aimed to investigate sex differences in HRQoL in MI survivals, the longitudinal trajectories of HRQoL over a 12-month period, and determine to what extent sex itself might explain the differences in HRQoL when accounting for other important factors.

Methods

Design and setting

Linked data from the Evaluation of the Methods and Management of Acute Coronary Events (EMMACE 3 and 4)[14] and Myocardial Ischaemia National Audit Project (MINAP)[15] were used for the analyses. The EMMACE studies are multi-centre nationwide longitudinal cohort studies of patients hospitalised with ACS. Patients were eligible for the study if they were ≥18 years of age. HRQoL data for MI survivors from 77 hospitals in England between 1 November 2011 and 24 June 2015 were collected at hospital admission (baseline), and longitudinally at 1, 6 and 12 months via questionnaires. Patients were consented for data linkage with MINAP to obtain information on the type of MI, baseline co-morbidities and in-hospital treatments. Fifteen participants (0.2%) had missing sex data and were excluded from the study. Of the 9,551 participants 35.7% (3,413) completed and returned the questionnaires at all-time points data was collected.

Assessment of HRQoL

EuroQol five dimension (EQ-5D-3L) questionnaire was used to collect HRQoL data[16].

The EQ-5D-3L descriptive system and EQ VAS were used in this study because the measures have previously been validated in MI patients and were found to be a valid general HRQoL measurement scale post MI [17]. Furthermore, this generic measure enables comparison of health problems among patients in different National Quality Registries, to understand the

For peer review only - http://bmjopen.bmj.com/site/about/guidelines.xhtml

overall severity of problems experienced by patients with different diseases and treatment pathways[18].

The EQ-5D questionnaire consists of questions covering five health domains, which include mobility, self-care, usual activities, pain/ discomfort and anxiety/ depression. An EQ-5D single score is derived based on these five dimensions taking into account societal preference weights [19]. The EQ-5D-3L profiles for each patient were combined with health state preference values from the UK general population to give EQ-5D-3L health state index scores ranging from −0.5 to 1, with scores less than 0 indicating states ‘worse than death’, 0 indicating no quality of life or ‘death’ and 1 indicating full health and therefore no problems in any domain. The index score has been standardised to the UK population and validity of the questionnaire in MI patients has been determined[16 19 20]. The questionnaire also has a visual analogue scale (EQ VAS) that allows participants to rate their current health state. The EQ VAS score ranges from 0 to 100 with 0 denoting the worst imaginable health state and 100 the best imaginable health state. A difference in the score of 7 for EQ VAS and 0.05 for EQ-5D is regarded as the minimal clinically important difference (MCID)[21].

Statistical analyses

Differences in baseline characteristics for men and women were described using frequencies and proportions for categorical data, means and standard deviations (SD) for normally distributed continuous data and medians and interquartile ranges (IQR) for non-normally distributed data. Multi-level linear regression was used to assess sex differences in HRQoL (EQ-5D and EQ VAS scores) in MI survivors. As the HRQoL data consisted of repeated measures nested within individuals and individuals nested within hospitals, the multi-level approach was implemented. Inverse probability weighted propensity scoring was used to weight the data and balance out systematic differences in baseline characteristics between

For peer review only

174 men and women to minimise selection bias (See Supplementary methods section 1, which
175 gives further detail of the methods). The primary outcomes of the study were the EQ-5D and
176 EQ VAS scores – with further subgroup analyses conducted for each of the EQ-5D domains
177 (mobility, self-care, usual activities, pain/discomfort and anxiety/ depression) using multi-
178 level logistic regression models. The domains are recorded as three level variables, however,
179 for this study they were treated as binary variables, 'some problems' and 'extreme problems'
180 levels vs. 'no problems'. The "extreme problem" category of the EQ-5D measure was
181 endorsed by few individuals for some domains (e.g. self-care and mobility) therefore we
182 combined the EQ-5D levels 'some problems' and 'extreme problems'.
183 To mitigate residual confounding the multi-level linear and logistic regression models were
184 adjusted for covariates which included aspirin, β-blockers, statins, angiotensin-converting
185 enzyme (ACE) inhibitor or angiotensin receptor blocker (ARB) and $P2Y_{12}$ inhibitors
186 prescription at hospital discharge, type of MI, enrolment into cardiac rehabilitation, coronary
187 intervention, body mass index (BMI), previous MI, age, index of multiple deprivation (IMD)
188 score, previous coronary artery bypass graft (CABG) surgery, smoking status, previous
189 percutaneous coronary intervention (PCI), family history of coronary heart disease (CHD),
190 peripheral vascular disease, hypercholesterolemia, previous angina, chronic obstructive
191 pulmonary disease (COPD) or asthma, diabetes mellitus, chronic renal failure, hypertension,
192 cerebrovascular disease and heart failure. Effect sizes were estimated evaluating changes in
193 HRQoL over time, i.e. from time of hospitalisation with MI (baseline) to 12 months post
194 hospital discharge. An interaction term of time and sex was added to the models to test if
195 there were significant sex differences in rate of improvement in HRQoL following AMI. The
196 models were fitted on the weighted balanced data.
197

For peer review only - http://bmjopen.bmj.com/site/about/guidelines.xhtml

Multiple imputation by chained equations[22] was used to impute missing data for the following variables: age, IMD score and BMI (See Supplementary Table 1, section 2, which gives detail of the imputation strategy used). Based on clinical expert opinion select binary treatment and medical history variables were imputed to 'no' if missing (See Supplementary Table 1, section 2, which gives detail of the imputation strategy used)[22]. Rubin's rules [23] were used to pool the results estimates and generate 95% confidence intervals. On non-weighted data predictors of change in HRQoL were explored by sequentially adding covariates (baseline HRQoL patient reported measures and patient baseline characteristics) to the bivariate multi-level linear regression model with sex only. Covariates which attenuated the sex differences in change in HRQoL observed were considered as predictors. Analysis were performed using Stata MP64 version 14 (StataCorp,www.stata.com) and R version 3.1.2. P-values <0.05 were considered statistically significant.

Patient and public involvement

The Leeds Teaching Hospitals (LTH) NHS Trust Cardiovascular Patient and Public Involvement group was involved in the project design. We also worked closely with a patient (GO) outside the group for the interpretation of the research findings, critical review of the manuscript and its dissemination.

Results

Study sample

At baseline (admission) a total of 9,551 patients with MI and complete data on sex, 25.1% (2,397) were females and the average age of the sample was 64.1 years, SD (11.95).

A total of 3,413 had complete follow up data at all-time points, 24.6% (841) were females and 75.36% (2,572) men. Characteristics of patients with missing follow-up data at one or

For peer review only

more time points versus those with compete follow up data at all-time points are presented in Supplementary Table 2. Patients with missing follow up data were younger, more likely to live in deprived areas as shown by higher IMD score, more likely to have more risk factors and comorbidities (smoking, diabetes, heart failure, chronical renal failure, COPD, Previous AMI).

At baseline, compared with men, women were older (mean age 67.1 [SD 12.0] years vs. 63.1 [SD 11.7] years), more likely to have hypertension (51.6 vs. 42.7%), COPD/asthma (16.3 vs. 11.7%), and to present with NSTEMI (62.9 vs. 57.9%) (Table 1). Conversely, men were more frequently smokers (68.9 vs. 62.2%), had higher rates of previous MI (17.6 vs. 14.3%), previous PCI (10.5 vs. 8.1%), or previous coronary artery bypass graft (CABG) surgery (7.9 vs. 4.7%), and were more likely to undergo coronary intervention during the hospital stay (48.6 vs. 41.6%) compared with women.

Table 1 Patient baseline characteristics, stratified by sex

Variables	Men n=7,154	Women n=2,397	P value	Missing[¥]
NSTEMI, n. (%)	4,141 (57.9)	1,507 (62.9)	<0.001	0
Age, mean (SD), yr.	63.1 (11.7)	67.1 (12.0)	<0.001	19 (0.2)
White ethnicity, n. (%)	6,027 (96.9)	2,099 (98.5)	<0.001	1,197 (12.5)
IMD, median (IQR)	17.9 (10.7-31.4)	20.5 (11.8-33.4)	<0.001	5,258 (55.1)
BMI, mean(SD), kg/ m^2	28.6 (6.1)	28.9 (6.0)	0.151	3,366 (35.2)
Previous angina, n. (%)	1,339 (19.7)	451 (19.9)	0.826	493 (5.2)
Diabetes, n. (%)	1,256 (18.2)	457 (19.6)	0.133	329 (3.4)
Hypertension, n. (%)	2,904 (42.7)	1,170 (51.6)	<0.001	487 (5.1)
Heart failure, n. (%)	150 (2.2)	62 (2.7)	0.151	503 (5.3)
Peripheral vascular disease, n. (%)	238 (3.6)	79 (3.6)	0.992	626 (6.6)
Cerebrovascular disease, n. (%)	305 (4.5)	123 (5.4)	0.065	496 (5.2)
Chronical renal failure, n. (%)	203 (3.0)	86 (3.8)	0.057	499 (5.2)

For peer review only - http://bmjopen.bmj.com/site/about/guidelines.xhtml

Variables	Men n=7,154	Women n=2,397	P value	Missing¥
COPD/ asthma, n. (%)	796 (11.7)	370 (16.3)	<0.001	429 (4.5)
Smoker and ex-smoker, n. (%)	4,786 (68.9)	1,456 (62.2)	<0.001	263 (2.8)
CABG surgery, n. (%)	534 (7.9)	107 (4.7)	<0.001	496 (5.2)
Previous PCI, n. (%)	713 (10.5)	184 (8.1)	<0.001	510 (5.3)
Previous MI, n. (%)	1,196 (17.6)	324 (14.3)	0.0003	486 (5.1)
Cardiac rehabilitation† (n=9,307), n. (%)	6,387 (97.7)	2,110 (97.5)	0.565	607 (6.4)
Coronary intervention† (n=8,859) (PCI/CABG), n. (%)	2,810 (48.6)	826 (41.6)	<0.001	1,094 (12.4)
**Discharge medications†**				
Beta-blocker (n=8,029), n. (%)	5,691 (98.4)	1,888 (98.0)	0.166	322 (4.0)
ACE or ARB inhibitor (n=8,134), n. (%)	5,727 (97.8)	1,871 (97.0)	0.051	348 (4.3)
Statin (n=8,520), n. (%)	6,118 (99.1)	2,009 (98.9)	0.265	317 (3.7)
Aspirin (n=8,499), n. (%)	6,107 (99.4)	2,026 (99.0)	0.048	308 (3.6)
$P2Y_{12}$ inhibitors (n=5,491), n. (%)	3,610 (97.6)	1,259 (96.5)	0.037	486 (8.9)

Abbreviation: ACEi – angiotensin-converting enzyme inhibitor; ACS – Acute coronary syndrome; ARBs – Angiotensin receptor blocker, IMD indicates Index of Multiple Deprivation; CABG, coronary artery bypass graft; PCI, percutaneous coronary intervention; MI, Myocardial Infarction; COPD, chronic obstructive pulmonary disease; BMI, body mass index; NSTEMI, non ST-elevation myocardial infarction.¥15 (0.2%) patients had missing sex data. † Only patients eligible to receive treatments were included in the denominator of the complete cases.

Patterns of HRQoL

At baseline women had lower HRQoL compared with men: EQ VAS mean (SD) 59.8 (20.4) and vs. 64.5 (20.9), EQ VAS median (IQR) 60.00 (50.00 to 75.00) vs. 70.00 (50.00 to 80.00) and EQ-5D mean (SD) 0.66 (0.31) and vs. 0.74 (0.28), EQ-5D median (IQR) 0.73 (0.52 to 0.85) vs. 0.81 (0.62 to 1.00). The observed difference persisted through all-time points of follow-up (Figure 1). Over time HRQoL improved for both men and women following MI (Figure 1). Compared with men, women were more likely to report problems in all dimensions of EQ-5D (Figure 2). In the first month for both men and women, there was an increase in the proportion of patients reporting problems with usual activities, pain/discomfort and anxiety/depression (Figure 2). However, improvements were observed in

For peer review only

the following months, with proportions of patients reporting pain/discomfort remaining stagnant (Figure 2).

Adjusted sex differences in HRQoL

The standardised differences showed that the weighting using the propensity scores balanced the systematic differences in baseline characteristics between men and women as the standardised differences were close or equal to zero (See Supplementary Table 3, which gives detail of the standardised differences). The minimum propensity score for each level was sufficiently greater than zero and that the maximum propensity score for each level was sufficiently less than 1, showing that the overlap assumption was not violated (Supplementary Figure 1). Compared with men, women had on average a lower HRQoL (adjusted EQ VAS model sex coefficient: -4.41, 95% CI -5.16 to -3.66 and adjusted EQ-5D model sex coefficient: -0.07, -0.08 to -0.06) and higher odds of reporting problems across all individual EQ-5D dimensions (Table 2). The interaction term exploring sex-based differences in the rates of HRQoL changes was not significant.

Table 2 Propensity score analysis to show health-related quality of life differences between women vs. men

**Health related quality of life**	**Coefficient (95% CI)**	**P value**
EQ VAS model		
Sex (Women vs. men)	-4.41 (-5.16 to -3.66)	<0.001
EQ 5D model		
Sex (Women vs. men)	-0.07 (-0.08 to -0.06)	<0.001
**EQ 5D dimensions**	**Odds ratio (95% CI)**	
Mobility problems model		
Sex (Women vs. men)	1.82 (1.58 to 2.09)	<0.001
Activities of daily living problems model		
Sex (Women vs. men)	1.70 (1.52 to 1.89)	<0.001

For peer review only - http://bmjopen.bmj.com/site/about/guidelines.xhtml

Self-care problems model		
Sex (Women vs. men)	1.75 (1.47 to 2.08)	<0.001
Pain/ discomfort model		
Sex (Women vs. men)	1.59 (1.45 to 1.75)	<0.001
Anxiety/ depression model		
Sex (Women vs. men)	2.03 (1.80 to 2.29)	<0.001

Factors associated with sex differences in HRQoL

Sex differences were observed in HRQoL in the bivariate model (EQ VAS model sex coefficient: -3.78, 95% CI -4.65 to -2.91 and EQ-5D model sex coefficient: -0.07, -0.08 to -0.06) (Table 3). The sex effect was markedly attenuated after accounting for patients' baseline HRQoL scores (EQ VAS coefficient: -2.56, 95% CI -3.38 to -1.73) (Table 3). However, for EQ-5D baseline scores did not attenuate the sex effect observed.

Table 3 Factors explaining sex differences observed in health related quality of life following MI

**Parameter**	**EQ-5D model, coefficient (95% CI)**	**EQ VAS model, coefficient (95% CI)**
Sex effect	-0.07 (-0.08 to -0.06)	-3.78 (-4.65 to -2.91)
Adding age, BMI, IMD	-0.06 (-0.07 to -0.05)	-3.33 (-4.20 to -2.40)
Adding pharmacotherapy and coronary intervention	-0.06 (-0.08 to -0.05)	-3.27 (-4.16 to -2.27)
Adding final diagnosis	-0.06 (-0.08 to -0.05)	-3.17 (-4.06 to -2.27)
Adding comorbidities and risk factors¥	-0.07 (-0.08 to -0.06)	-3.77 (-4.64 to -2.90)
Adding baseline value of the HRQoL metric*	-0.06 (-0.07 to -0.05)	-2.56 (-3.38 to -1.73)

Abbreviations: * Baseline EQ-5D for EQ-5D model and baseline EQVAS for EQ VAS model. BMI, body mass index; IMD indicates Index of Multiple Deprivation; MI, Myocardial Infarction; HRQoL, health related quality of life. ¥ Previous MI, age, previous coronary artery bypass graft surgery, smoking status, previous percutaneous coronary intervention, family history of coronary heart disease , peripheral vascular disease, hypercholesterolemia, previous angina, chronic obstructive pulmonary disease or asthma, diabetes mellitus, chronic renal failure, hypertension, cerebrovascular disease and heart failure.

For peer review only - http://bmjopen.bmj.com/site/about/guidelines.xhtml

Discussion

In this national longitudinal cohort study of 9,551 consecutive patients hospitalised with MI, we demonstrated that 1) women had lower HRQoL compared with men at baseline and throughout the following 12 months; 2) trajectories in HRQoL scores and all EQ-5D-3L domains (mobility, personal care, activities of daily living, pain/discomfort and anxiety/depression) assessed at 4 time points were similar between groups; and 3) adjustment for other variables, including age, risk factors, comorbidity, treatment, final diagnosis and baseline HRQoL decreased but did not eliminate the differences observed in HRQoL in women and men following MI.

To our knowledge, we present the largest longitudinal study to assess sex differences in MI survivors. Prior studies have addressed this question, however, they have been limited to small sample sizes[6-10], sub-selecting only MI patients receiving certain interventions [11], and short follow-up [11 24]. Data on 12-months HRQoL trajectories from contemporary real-world patient populations is limited.

Similar to our findings, previous research has shown that women report lower HRQoL at time of their presentation with MI[13 25], but the sex differences in baseline health status prior to MI have been attributed to the fact that women usually report more mental health disorders such as depression, fatigue or anxiety compared with men [26-28]. Our study, similarly to a recent large study of contemporary ACS patients treated with PCI, found that female sex was independently associated with significant impairment in all EQ-5D-3L domains (mobility, personal care, activities of daily living, pain/discomfort and anxiety/depression) [11]. Moreover, during longitudinal 12-months assessment women consistently reported lower HRQoL as measured by overall EQ-5D score, EQ VAS and problems at each of EQ-5D domains.

For peer review only - http://bmjopen.bmj.com/site/about/guidelines.xhtml

Between sex differences in epidemiology, pathophysiology, risk factors, clinical presentation and treatment strategies which have been demonstrated for MI patients are likely contributing to the observed differences in HRQoL following MI. Coronary revascularisation after MI was associated with improvements in HRQoL for both men and women, yet similarly to prior findings in our study women less frequently underwent coronary intervention. In recent years, an increasing emphasis has been placed on the association between multi-morbidity level and negative outcomes in MI survivors. Indeed, the changes in HRQoL in men have been found to be associated with presenting characteristics of MI and complications of treatment while those of women were linked with their demographic characteristics and comorbidities[13 29]. In our study women were older and had higher premorbid conditions at baseline, the presence of which has been associated with worst HRQoL following MI. Importantly however, after adjustment for multiple confounders, including comorbidities and treatment strategies such as medication, revascularization and cardiac rehabilitation, between sex differences in HRQoL remained significant for our study. Another study though suggested an impact of sex on physical functioning only, while gender-related factors such as femininity score, social support, and housework responsibility are independent predictors of long-term HRQoL[30]. Recognition of the associations of sex and gender itself with a diverse spectrum of factors related to cardiovascular and general health has led to a recently proposed concept of sex-and gender-sensitive medicine. Still translation of this concept into routine clinical care is far from desired. Our study further magnifies this by demonstrating the lack of trend towards closing the between-sex gap during 12 months follow-up after MI in the large clinical practice-based patient population data from the EMMACE studies. From clinical medicine and physicians' perspective, in order to reduce the existing disparities, attempts should be sought to improve women's health status by identification of as many potential reasons as possible, addressing the modifiable risk factors and engaging more women into the

For peer review only - http://bmjopen.bmj.com/site/about/guidelines.xhtml

339 recommended multidisciplinary post-MI management programmes. Lack of systematic
340 assessment of gender-specific factors in many studies, including our study, highlight a need
341 for large-scale strategic initiatives from public health and social care to better understand and
342 support many intertwined factors affecting women health.
343 In our study the meaningful improvement in HRQoL has been demonstrated equally in both
344 men and women. Although our study is in line with a previous analysis of young MI
345 populations in terms of general positive HRQoL trend after MI independently of sex [31], in
346 other studies different patterns have been found for women mainly reporting an improvement
347 in mental functioning while men tend to report improvement in the physical health status [8 32].
348 Analysis of magnitude of change in health status showed the highest increment improvements
349 of EQ-5D and EQ VAS score at 1 month after discharge and it's plateauing after 6 months.
350 Counterintuitively however, at 1 month time point the highest proportions of patients
351 reported some problems in anxiety/depression, pain/discomfort and usual activities. This
352 highlights that measuring not only health status via serial general HRQoL assessments but
353 also its domains are needed. Moreover, considering independent relationship between EQ-5D
354 and its domains in MI survivors and mortality[1], this strategy might provide advantages in
355 identification of patients at the highest risk of negative outcomes. Indeed, analysis of 26,641
356 patients with first MI from SWEDEHEART registry have showed that anxiety/depression
357 assessed 6-10 weeks after MI is associated with 29% higher risk of cardiovascular mortality
358 and 34% higher risk of non-cardiovascular mortality independently of traditional risk factors.
359 Though these associations remained significant only if the mental problems persisted after 12
360 months[33]. Another multinational study of HRQoL as assessed by EQ-5D in 8978 post-MI
361 patients showed that the presence of problems on 'self-care' and 'mobility' were most
362 powerful predictors of all-cause mortality, whereas problems with pain/discomfort and usual
363 activities were most strongly associated with cardiovascular events[1].

For peer review only - http://bmjopen.bmj.com/site/about/guidelines.xhtml

Our findings can be interpreted using the Wilson Clearly HRQoL [34] conceptual model, which causally links five health concepts, the biological and physiological factors, symptoms, functional health, general health perceptions and HRQoL. Symptoms mediate between physiological factors and functional status; functional status mediates between symptoms and general health perceptions, and general health perceptions mediates between functional status and overall HRQL [35]. A systematic review found that more symptoms implied impaired functioning which may lead to worse general health perception and consequently lower HRQoL[35]. Compared to men, women reported more symptoms and problems with physiological factors and functional status and these potentially mediate between functional statuses which can have a direct effect on overall HRQoL.

Future large studies of sex- and gender-differences and effects of targeted interventions after MI might help to further personalize management strategies and as a result improve HRQoL outcomes in women.

Implications of the study

Our findings build on other previous studies suggesting lower HRQoL in women compared to men, but strengthen them by reporting absence of significant difference in patterns of changes throughout 12 months follow-up in a broad real-world MI population. Higher adoption of serial assessment of patient-reported outcomes such as HRQoL are needed to tailor treatment interventions. The quantification of HRQoL at time of MI and identification of predictors of recovery may be important for designing targeted interventions tailored to meet the needs of patients and improve their physical and mental wellbeing.

For peer review only - http://bmjopen.bmj.com/site/about/guidelines.xhtml

Strengths and limitations

Our study has strengths in that it evaluates changes in HRQoL using nationwide longitudinal data which minimises selection bias and increases generalisability. There are no other databases of comparable size, coverage and quality. An inverse weighted propensity scoring approach was applied to weight data and balance out systematic differences based on observed covariates to minimise inherent bias. However, our study has limitations. 1) We used a generic quality of life metric rather than a disease-specific one to measure HRQoL. Nonetheless, the EQ-5D does capture dimensions of the quality of life that are relevant to, and are impacted by, MI such as mobility, depression/anxiety and pain/discomfort. In addition, EQ-5D has been validated in MI patients, and using a generic metric allows the comparison for the magnitude of HRQoL impairment between MI and other diseases. More work is required using causal HRQoL conceptual frameworks such as the Wilson Clearly causal framework [34] to gain a deeper understanding of the nature of HRQoL and factors contributing to it. 2) The generalisability of the study's findings may be limited by a selection bias inherent as a result of loss to follow-up data. However, sensitivity analyses comparing those lost follow-up with those who were not showed minimal systematic differences (See Supplementary Table 2, which compares baseline characteristics of patients with complete follow-up with those missing one or more follow-up data points). 3) Similar to many large cardiovascular registries, our study did not collect sex-specific characteristics, particularly social status and mental disorders prior to MI, therefore there could be residual confounding. However, we adjusted for an extensive range of patient level factors that are usually included in similar sex based research including IMD which is a measure of relative deprivation derived from combining 7 domains: income, employment, education, skills and training, health and disability, crime to housing services, living environment".

For peer review only - http://bmjopen.bmj.com/site/about/guidelines.xhtml

Conclusion

In this national longitudinal study, women have lower HRQoL by index EQ-5D score at baseline and during 12 months follow-up after MI and persistently reported higher levels of impairment in their mobility, self-care, usual activities, pain/discomfort and anxiety/ depression at each time point assessment. The magnitude of HRQoL improvement was similar between groups. Sex differences were attenuated by baseline HRQoL scores. Targeted interventions to address the reasons behind poor baseline health status, particularly gender-specific factors and multiple domains of HRQoL, could improve health outcomes in women after MI. During cardiac rehabilitation following MI, EQ-5D can be used as a tool to identify women at risk of poor HRQoL and dimensions mostly affected allowing targeted intervention.

Acknowledgements

We gratefully acknowledge the contributions from all hospitals, healthcare professionals and patients who participated in the EMMACE study. We would also like to thank Richard Gillott (IT support), Claire Forrest (Cardiology Research Coordinator) and Vera Hall (Finance support).

For peer review only - http://bmjopen.bmj.com/site/about/guidelines.xhtml

Contributorship statement

TBD analysed the data and drafted the manuscript. CPG and ASH contributed to the conception of the research, funding acquisition, project administration, supervision, study design and data collection. CPG, ASH, SA, AS, and BH provided expert clinical opinion and interpretation of the data. RMW, MH, TM and TBD provided statistical expert advice and interpretation of the data. GO was involved as a patient advisor in the interpretation of the research and the writing of the manuscript. All authors made critical revisions and provided intellectual content to the manuscript, approved the final version to be published and agreed to be accountable for all aspects of the work.

Competing interests

CPG reports personal fees from AstraZeneca, personal fees from Bayer, personal fees from Boehringer Inglehiem, personal fees from Amgen, personal fees from Daiichi Sankyo, personal fees from Vifor Pharma, grants from Abbott, grants from BMS, outside the submitted work. BH reports grants from National Institute for Health Research (NIHR/CS/009/004) and British Heart foundation (PG/19/54/34511), during the conduct of the study. AH reports personal fees (speaker honorarium) from NOVARTIS & SERVIER. AS acknowledges funding received from the European Society of Cardiology in form of an ESC Research Grant. The remaining authors have nothing to disclose. All authors have completed the ICMJE uniform disclosure form at www.icmje.org/coi_disclosure.pdf.

Funding

This work was supported by the National Institute for Health Research (NIHR/CS/009/004), and British Heart Foundation (PG/19/54/34511). MH was funded by the Wellcome Trust (grant reference: reference: 206470/Z/17/Z).

For peer review only

Data sharing statement

The datasets used and/or analysed during the current study are available from the principal investigator of the study on reasonable request.

Ethics

EMMACE-3 has been given a favourable ethical opinion by the Leeds (Central) Research Ethics committee (REC reference: 10/H1313/74), is registered on ClinicalTrials.gov (NCT01808027) and has been adopted onto the National Institute for Health Research Comprehensive Research Network portfolio (9102). EMMACE-4 has been given favourable ethical opinion by the West Midlands - Black Country Research Ethics Committee (REC reference: 12/WM/0431), is registered on ClinicalTrials.gov (NCT01819103) and has been adopted onto the National Institute for Health Research Comprehensive Research Network portfolio (9102)

Word count: Abstract: 295

Main text excluding tables, figures and references: 3,514

Tables: 3

Figures: 2

For peer review only - http://bmjopen.bmj.com/site/about/guidelines.xhtml

References

1. Pocock S, Brieger DB, Owen R, et al. Health-related quality of life 1–3 years post-myocardial infarction: its impact on prognosis. Open Heart 2021;**8**(1):e001499.

2. Dreyer RP, Ranasinghe I, Wang Y, et al. Sex differences in the rate, timing, and principal diagnoses of 30-day readmissions in younger patients with acute myocardial infarction. Circulation 2015;**132**(3):158-66.

3. Dreyer RP, Dharmarajan K, Kennedy KF, et al. Sex differences in 1-year all-cause rehospitalization in patients after acute myocardial infarction: a prospective observational study. Circulation 2017;**135**(6):521-31.

4. Vaccarino V, Rathore SS, Wenger NK, et al. Sex and racial differences in the management of acute myocardial infarction, 1994 through 2002. N Engl J Med 2005;**353**(7):671-82.

5. Canto JG, Rogers WJ, Goldberg RJ, et al. Association of age and sex with myocardial infarction symptom presentation and in-hospital mortality. JAMA 2012;**307**(8):813-22.

6. Emery CF, Frid DJ, Engebretson TO, et al. Gender differences in quality of life among cardiac patients. Psychosom Med 2004;**66**(2):190-97.

7. Beck CA, Joseph L, Bélisle P, et al. Predictors of quality of life 6 months and 1 year after acute myocardial infarction. Am Heart J 2001;**142**(2):271-79.

8. Brink E, Grankvist G, Karlson BW, et al. Health-related quality of life in women and men one year after acute myocardial infarction. Qual Life Res 2005;**14**(3):749-57.

9. Lacey E, Walters S. Continuing inequality: gender and social class influences on self perceived health after a heart attack. J Epidemiol Community Health 2003;**57**(8):622-27.

10. Worcester MUC, Murphy BM, Elliott PC, et al. Trajectories of recovery of quality of life in women after an acute cardiac event. Br J Health Psychol 2007;**12**(1):1-15.

11. Koh Y, Stehli J, Martin C, et al. Does sex predict quality of life after acute coronary syndromes: an Australian, state-wide, multicentre prospective cohort study. BMJ open 2019;**9**(12).

12. Dreyer RP, Wang Y, Strait KM, et al. Gender differences in the trajectory of recovery in health status among young patients with acute myocardial infarction: results from the variation in recovery: role of gender on outcomes of young AMI patients (VIRGO) study. Circulation 2015;**131**(22):1971-80.

13. Dueñas M, Ramirez C, Arana R, et al. Gender differences and determinants of health related quality of life in coronary patients: a follow-up study. BMC Cardiovasc Disord 2011;**11**(1):24.

For peer review only

For peer review only - http://bmjopen.bmj.com/site/about/guidelines.xhtml

14. Alabas O, West R, Gillott R, et al. Evaluation of the Methods and Management of Acute Coronary Events (EMMACE)-3: protocol for a longitudinal study. BMJ open 2015;**5**(6):e006256.

15. Herrett E, Smeeth L, Walker L, et al. The Myocardial Ischaemia National Audit Project (MINAP). Heart 2010;**96**(16):1264-7.

16. Cheung K, Oemar M, Oppe M, et al. EQ-5D user guide: basic information on how to use EQ-5D. Rotterdam: EuroQol Group 2009.

17. Nowels D, McGloin J, Westfall JM, et al. Validation of the EQ-5D quality of life instrument in patients after myocardial infarction. Quality of life research 2005;**14**(1):95-105.

18. Teni FS, Rolfson O, Devlin N, et al. Longitudinal study of patients' health-related quality of life using EQ-5D-3L in 11 Swedish National Quality Registers. BMJ open 2022;**12**(1):e048176.

19. Dolan P. Modeling valuations for EuroQol health states. Med care 1997:1095-108.

20. Nowels D, McGloin J, Westfall JM, et al. Validation of the EQ-5D quality of life instrument in patients after myocardial infarction. Qual Life Res 2005;**14**(1):95-105.

21. Nolan CM, Longworth L, Lord J, et al. The EQ-5D-5L health status questionnaire in COPD: validity, responsiveness and minimum important difference. Thorax 2016;**71**(6):493-500.

22. Cattle B, Baxter P, Greenwood D, et al. Multiple imputation for completion of a national clinical audit dataset. Stat Med 2011;**30**(22):2736-53.

23. Rubin DB. *Multiple imputation for nonresponse in surveys*: John Wiley & Sons, 2004.

24. Failde II, Soto MM. Changes in health related quality of life 3 months after an acute coronary syndrome. BMC Public Health 2006;**6**(1):18.

25. Norris CM, Spertus JA, Jensen L, et al. Sex and gender discrepancies in health-related quality of life outcomes among patients with established coronary artery disease. Circ Cardiovasc Qual Outcomes 2008;**1**(2):123-30.

26. Dreyer RP, Smolderen KG, Strait KM, et al. Gender differences in pre-event health status of young patients with acute myocardial infarction: A VIRGO study analysis. Eur Heart J Acute Cardiovasc Care 2016;**5**(1):43-54.

27. Barnett K, Mercer SW, Norbury M, et al. Epidemiology of multimorbidity and implications for health care, research, and medical education: a cross-sectional study. Lancet 2012;**380**(9836):37-43.

28. Parashar S, Rumsfeld JS, Reid KJ, et al. Impact of depression on sex differences in outcome after myocardial infarction. Circ Cardiovasc Qual Outcomes 2009;**2**(1):33-40.

29. Höfer S, Benzer W, Oldridge N. Change in health-related quality of life in patients with coronary artery disease predicts 4-year mortality. Int J Cardiol 2014;**174**(1):7-12.

For peer review only - http://bmjopen.bmj.com/site/about/guidelines.xhtml

30. Leung Yinko SS, Pelletier R, Behlouli H, et al. Health-Related quality of life in premature acute coronary syndrome: does patient sex or gender really matter? J Am Heart Assoc 2014;**3**(4):e000901.

31. Dreyer RP, Zheng X, Xu X, et al. Sex differences in health outcomes at one year following acute myocardial infarction: a report from the China Patient-Centered Evaluative Assessment of Cardiac Events prospective acute myocardial infarction study. Eur Heart J Acute Cardiovasc Care 2019;**8**(3):273-82.

32. Staniūtė M, Brožaitienė J. Changes in health-related quality of life among patients with coronary artery disease: a 2-year follow-up. Medicina 2010;**46**(12):843.

33. Lissåker CT, Norlund F, Wallert J, et al. Persistent emotional distress after a first-time myocardial infarction and its association to late cardiovascular and non-cardiovascular mortality. Eur J Prev Cardiol 2019;**26**(14):1510-18.

34. Wilson IB, Cleary PD. Linking clinical variables with health-related quality of life: a conceptual model of patient outcomes. Jama 1995;**273**(1):59-65.

35. Ojelabi AO, Graham Y, Haighton C, et al. A systematic review of the application of Wilson and Cleary health-related quality of life model in chronic diseases. Health and quality of life outcomes 2017;**15**(1):1-15.

For peer review only

For peer review only - http://bmjopen.bmj.com/site/about/guidelines.xhtml

Figure Legends

Figure 1 Health related quality of life trajectories following myocardial infarction by sex and UK general population

Figure 2 Health related quality of life domains trajectories following myocardial infarction by sex

For peer review only

For peer review only - http://bmjopen.bmj.com/site/about/guidelines.xhtml

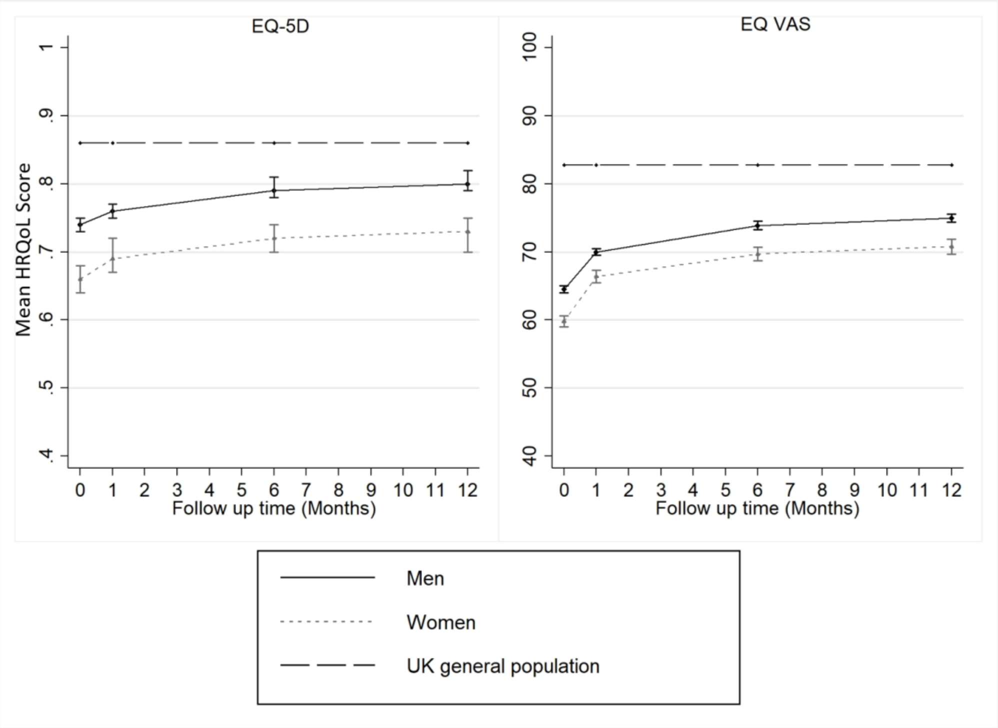

Figure 1

254x184mm (150 x 150 DPI)

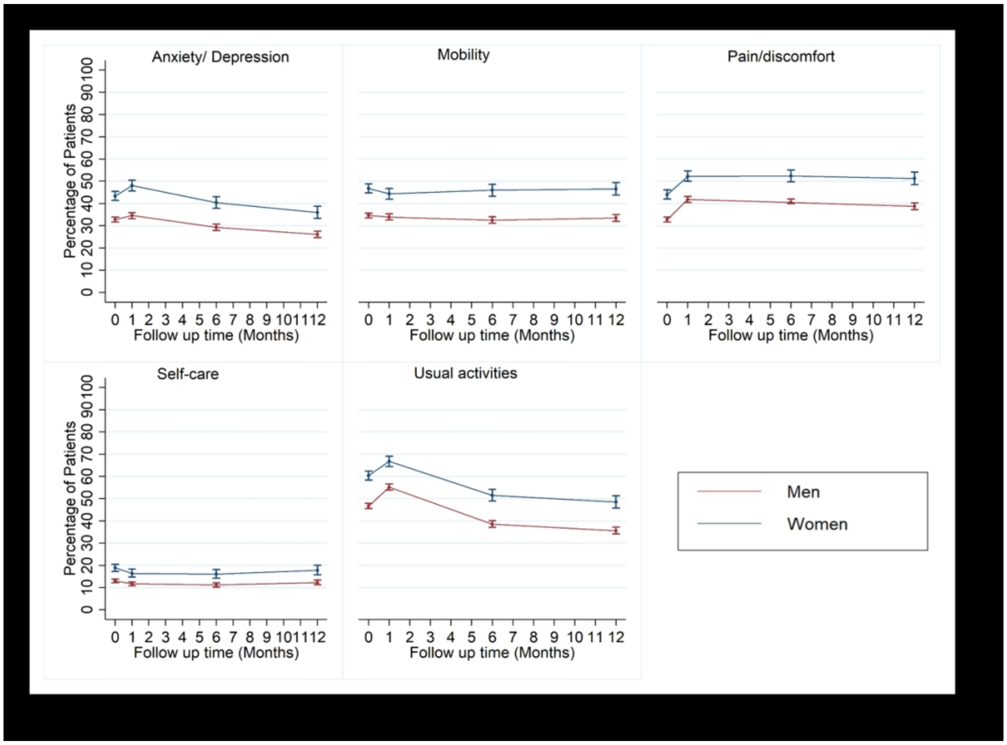

Figure 2

227x167mm (150 x 150 DPI)

SUPPLEMENTARY MATERIAL

Title: Sex differences in health-related quality of life trajectories following acute myocardial infarction: National longitudinal cohort study.

Authors

T.B. Dondo (PhD), T. Munyombwe (PhD), M. Hall (PhD), B. Hurdus (MBBS), A.Soloveva (PhD)[1,2], G. Oliver (Patient), S. Aktaa (MD), RM West (DPhil), A.S. Hall (FRCP), C.P. Gale (PhD FRCP).

For peer review only

Section 1: Supplementary methods

Propensity score analysis

A non-parsimonious multivariable logistic regression model was used to derive propensity scores (PS) (probability of being a man or a woman, conditional on observed patient baseline covariates) to weight the data balancing out systematic differences. The model was adjusted for patient baseline characteristics; AMI phenotype (STEMI vs. NSTEMI), age, body mass index (BMI), index of multiple deprivation (IMD) score, smoking status (never vs. current or ex-smoker), family history of coronary heart disease (CHD), previous angina, history of diabetes mellitus, hypertension, heart failure, peripheral vascular disease, cerebrovascular disease, chronic renal failure, chronic obstructive pulmonary disease (COPD) or asthma, hypercholesterolemia, previous percutaneous coronary intervention (PCI), previous coronary artery bypass graft (CABG) surgery, previous AMI, coronary intervention, hospital discharge medications (aspirin, β blockers, statins, ACEi/ARB and $P2Y_{12}$ inhibitors) and referral for cardiac rehabilitation. In order to assess whether the weights constructed from the propensity score model balanced the covariates between men and women, standardised differences were derived, a perfectly balanced covariate has a standardised difference of zero. Violation of the overlap assumption was assessed using an overlap plot and by summarising the estimated probabilities of sex. Supplemental Figure 1 illustrates the results of the assessment of the overlap assumption and shows that the minimum propensity score for each level was sufficiently greater than zero and that the maximum propensity score for each level was sufficiently less than 1, thus the assumption was not violated. The area under the curve for the propensity score model was 0.64, which indicated moderate discrimination for the model.

For peer review only

Supplementary Figure 1. Overlap assumption assessment plots (A) and Distribution of propensity scores across comparison groups (B).

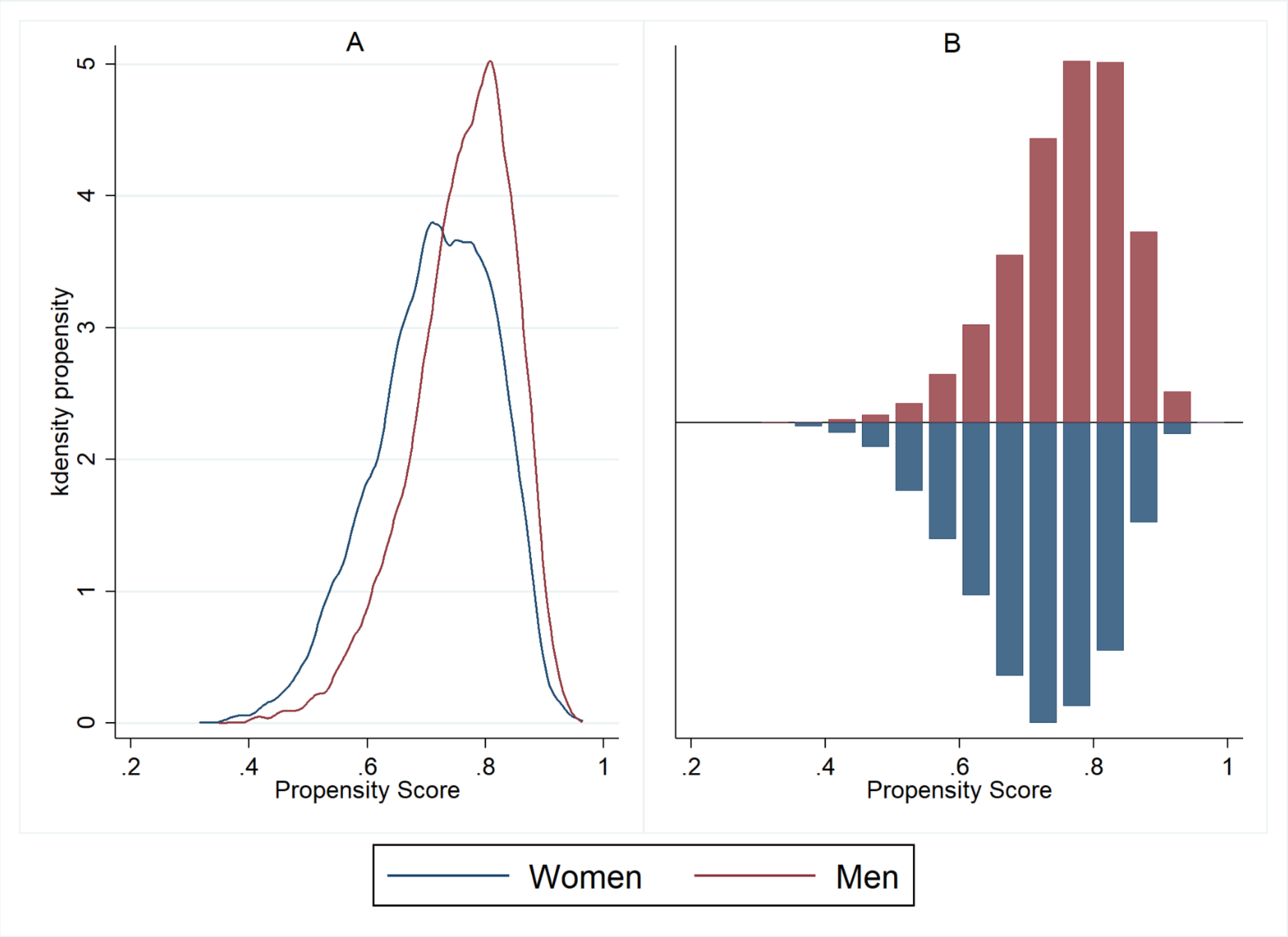

Section 2: Supplementary methods

Handling missing data in baseline characteristics

Missing data were imputed using multiple imputations by chained equations. Ten imputed datasets were derived from 20 iterations. Data were imputed for missing baseline characteristics and not follow-up outcome data. A default imputation (missing data default imputed to "NO") strategy based on clinical expert opinion was implemented for cardiovascular history, cardiovascular risk factors, and categorical treatment variables. The imputation strategy applied is summarised in Supplemental Table 1.

Supplementary Table 1. Imputation Strategy

Variable	Variable Type	Missing n (%)	Imputation Method
Sex	Binary	15 (0.16)	Predictor/ Auxiliary
Age	Continuous	19 (0.20)	Predictive mean matching
Family history of CHD	Binary	1,473 (15.40)	Predictor/ Auxiliary and Default imputed
Previous PCI	Binary	510 (5.33)	Predictor/ Auxiliary and Default imputed
Previous CABG	Binary	496 (5.19)	Predictor/ Auxiliary and Default imputed
Overallimdscore	Continuous	5,260 (55.00)	Predictive mean matching
BMI	Continuous	3,374 (35.27)	Predictive mean matching
Cardiac rehabilitation referral at hospital discharge	Binary	609 (6.37)	Predictor/ Auxiliary and Default imputed
Previous AMI	Binary	486 (5.08)	Predictor/ Auxiliary and Default imputed
Previous Angina	Binary	493 (5.15)	Predictor/ Auxiliary and Default imputed
Hypertension	Binary	487 (5.09)	Predictor/ Auxiliary and Default imputed
Hypercholesterolaemia	Binary	567 (5.93)	Predictor/ Auxiliary and Default imputed

For peer review only

Variable	Variable Type	Missing n (%)	Imputation Method
Peripheral Vascular Disease	Binary	626 (6.54)	Predictor/ Auxiliary and Default imputed
Cerebrovascular Disease	Binary	496 (5.19)	Predictor/ Auxiliary and Default imputed
Asthma or COPD	Binary	506 (5.29)	Predictor/ Auxiliary and Default imputed
Chronic Renal Failure	Binary	499 (5.22)	Predictor/ Auxiliary and Default imputed
Diabetes	Binary	330 (3.45)	Predictor/ Auxiliary and Default imputed
Reinfarction	Binary	510 (5.33)	Predictor/ Auxiliary and Default imputed
EQ-5D at admission	Continuous	366 (3.83)	Predictor/ Auxiliary
EQVAS at admission	Continuous	303 (3.17)	Predictor/ Auxiliary
Mobility	Binary	234 (2.45)	Predictor/ Auxiliary
Self-care	Binary	250 (2.61)	Predictor/ Auxiliary
Usual activities	Binary	300 (3.14)	Predictor/ Auxiliary
Pain/discomfort	Binary	250 (2.61)	Predictor/ Auxiliary
Anxiety/depression	Binary	252 (2.63)	Predictor/ Auxiliary

Abbreviations: CABG, coronary artery bypass graft; PCI, percutaneous coronary intervention; MI, Myocardial Infarction; COPD, chronic obstructive pulmonary disease; BMI, body mass index; CHD, coronary heart disease.

For peer review only

Supplementary Table 2. Patient baseline characteristics (patients with complete follow-up data vs patients with missing follow-up data at one or time points).

**Variables**	**Patients with complete follow up data at all-time points (n= 3,413)**	**Patients with missing follow-up data at one or time points (n= 6,138)**	**P value**
NSTEMI, n. (%)	2,051 (60.1)	3,597 (58.6)	0.155
Age, mean (SD), yr.	65.9 (10.6)	63.1 (12.5)	<0.001
White ethnicity, n. (%)	2,915 (98.7)	5,211 (96.5)	<0.001
IMD, median (IQR)	16.3 (10.2-27.1)	20.6 (11.9-34.0)	<0.001
BMI, mean(SD), kg/ m^2	27.4 (24.9-30.8)	28.1 (25.1-31.6)	<0.001
Previous angina, n. (%)	619 (19.0)	1,171 (20.2)	0.193
Diabetes, n. (%)	506 (15.4)	1,207 (20.3)	<0.001
Hypertension, n. (%)	1,486 (45.7)	2,588 (44.5)	0.303
Heart failure, n. (%)	62 (1.9)	150 (2.6)	0.042
Peripheral vascular disease, n. (%)	97 (3.1)	220 (3.8)	0.056
Cerebrovascular disease, n. (%)	136 (4.2)	292 (5.0)	0.069
Chronical renal failure, n. (%)	76 (2.3)	213 (3.7)	<0.001
COPD, n. (%)	374 (11.5)	792 (13.7)	0.004
Smoker and ex-smoker, n. (%)	2,050 (62.0)	4,192 (70.1)	<0.001
CABG surgery, n. (%)	227 (7.0)	414 (7.1)	0.826
Previous PCI, n. (%)	300 (9.3)	597 (10.3)	0.119
Previous AMI, n. (%)	485 (14.9)	1,035 (17.8)	<0.001
Cardiac rehabilitation[†] (n=8,700), n. (%)	3,049 (97.8)	5,448 (97.6)	0.681
Coronary intervention[†] (n=7,261), n. (%)	1,563 (60.3)	2,765 (59.2)	0.353
**Discharge medications**[†]			

For peer review only

Variables	Patients with complete follow up data at all-time points (n= 3,413)	Patients with missing follow-up data at one or time points (n= 6,138)	P value
Beta-blocker (n=7,708), n (%)	2,741 (98.4)	4,838 (98.3)	0.764
ACEi or ARB inhibitor (n=7,786), n. (%)	2,742 (97.8)	4,856 (97.5)	0.467
Statin (n=8,203), n. (%)	2,911 (99.3)	5,216 (99.0)	0.214
Aspirin (n=8,191), n. (%)	2,920 (99.3)	5,213 (99.3)	0.822
$P2Y_{12}$ inhibitors (n=5,005), n. (%)	1,828 (97.4)	3,041 (97.2)	0.593

Abbreviations: ACEi – angiotensin-converting enzyme inhibitor; ACS – Acute coronary syndrome; ARBs – Angiotensin receptor blocker, IMD indicates Index of Multiple Deprivation; CABG, coronary artery bypass graft; PCI, percutaneous coronary intervention; MI, Myocardial Infarction; COPD, chronic obstructive pulmonary disease; BMI, body mass index; NSTEMI, non ST-elevation myocardial infarction.

For peer review only

Section 3: Supplementary Tables

The balance check results are summarised in Supplemental Table 3, which shows that the standardised differences for variables in the weighted data were close to zero. The diagnostic assessments suggest that weighting by the inverse probability of sex created a sample in which the distributions of the covariates were similar between men and women.

Supplementary Table 3. Covariate balance across men and women after weighting on the propensity score

Variable	Mean in men	Mean in women	Standardized difference
Age	62.28	65.62	-0.285
Deprivation (IMD score)	22.55	24.68	-0.135
BMI	28.46	29.05	-0.108
Smoker ever	0.69	0.64	0.118
Final diagnosis	0.44	0.41	0.058
Family history of CHD	0.40	0.40	0.003
Previous PCI	0.07	0.06	0.023
Previous CABG	0.06	0.04	0.092
Previous MI	0.13	0.09	0.132
Hypertension	0.43	0.53	-0.201
Hypercholesterolaemia	0.34	0.34	-0.008
PVD	0.03	0.04	-0.049
CVSD	0.04	0.04	0.038
COPD	0.11	0.16	-0.126
Chronic renal failure	0.02	0.04	-0.106
Chronic cardiac failure	0.01	0.01	0.033
Diabetes	0.15	0.17	-0.046
Discharge medications			
Aspirin	0.89	0.87	0.050
β blockers	0.83	0.83	-0.002
Statin	0.89	0.88	0.036
ACEi/ARBs	0.85	0.83	0.056
$P2Y_{12}$ inhibitors	0.78	0.77	0.015

For peer review only

Coronary intervention	0.52	0.45	0.139
Cardiac rehabilitation	0.93	0.93	0.005

Abbreviations: ACEi – angiotensin-converting enzyme inhibitor; ACS – Acute coronary syndrome; ARBs – Angiotensin receptor blocker, IMD indicates Index of Multiple Deprivation; CABG, coronary artery bypass graft; PCI, percutaneous coronary intervention; MI, Myocardial Infarction; COPD, chronic obstructive pulmonary disease; CVSD cerebrovascular disease; BMI, body mass index.

For peer review only

STROBE Statement—checklist of items that should be included in reports of observational studies

	Item No	Recommendation	Page number	
**Title and abstract**	1	(*a*) Indicate the study's design with a commonly used term in the title or the abstract	1	✓
		(*b*) Provide in the abstract an informative and balanced summary of what was done and what was found	2-3	✓
**Introduction**				
Background/rationale	2	Explain the scientific background and rationale for the investigation being reported	5-6	✓
Objectives	3	State specific objectives, including any prespecified hypotheses	5-6	✓
**Methods**				
Study design	4	Present key elements of study design early in the paper	6	✓
Setting	5	Describe the setting, locations, and relevant dates, including periods of recruitment, exposure, follow-up, and data collection	6	✓
Participants	6	(*a*) *Cohort study*—Give the eligibility criteria, and the sources and methods of selection of participants. Describe methods of follow-up<br>*Case-control study*—Give the eligibility criteria, and the sources and methods of case ascertainment and control selection. Give the rationale for the choice of cases and controls<br>*Cross-sectional study*—Give the eligibility criteria, and the sources and methods of selection of participants	6	✓
		(*b*) *Cohort study*—For matched studies, give matching criteria and number of exposed and unexposed<br>*Case-control study*—For matched studies, give matching criteria and the number of controls per case		NA
Variables	7	Clearly define all outcomes, exposures, predictors, potential confounders, and effect modifiers. Give diagnostic criteria, if applicable	6-8	✓
Data sources/ measurement	8*	For each variable of interest, give sources of data and details of methods of assessment (measurement). Describe comparability of assessment methods if there is more than one group	6-8	✓
Bias	9	Describe any efforts to address potential sources of bias	7-8	✓
Study size	10	Explain how the study size was arrived at	6	✓
Quantitative variables	11	Explain how quantitative variables were handled in the analyses. If applicable, describe which groupings were chosen and why	7	✓
Statistical methods	12	(*a*) Describe all statistical methods, including those used to control for confounding	7-8	✓
		(*b*) Describe any methods used to examine subgroups and interactions	8	✓
		(*c*) Explain how missing data were addressed	9	✓
		(*d*) *Cohort study*—If applicable, explain how loss to follow-up was addressed<br>*Case-control study*—If applicable, explain how matching of cases and controls was addressed		N/A

For peer review only - http://bmjopen.bmj.com/site/about/guidelines.xhtml

		Cross-sectional study—If applicable, describe analytical methods taking account of sampling strategy		
		(*e*) Describe any sensitivity analyses	N/A	

Continued on next page

For peer review only

**Results**				
Participants	13*	(a) Report numbers of individuals at each stage of study—eg numbers potentially eligible, examined for eligibility, confirmed eligible, included in the study, completing follow-up, and analysed	9	
		(b) Give reasons for non-participation at each stage	-	
		(c) Consider use of a flow diagram	-	
Descriptive data	14*	(a) Give characteristics of study participants (eg demographic, clinical, social) and information on exposures and potential confounders	9-10	✓
		(b) Indicate number of participants with missing data for each variable of interest	9-10	✓
		(c) *Cohort study*—Summarise follow-up time (eg, average and total amount)	11	✓
Outcome data	15*	*Cohort study*—Report numbers of outcome events or summary measures over time	11	✓
		Case-control study—Report numbers in each exposure category, or summary measures of exposure		
		Cross-sectional study—Report numbers of outcome events or summary measures		
Main results	16	(*a*) Give unadjusted estimates and, if applicable, confounder-adjusted estimates and their precision (eg, 95% confidence interval). Make clear which confounders were adjusted for and why they were included	11-13	✓
		(*b*) Report category boundaries when continuous variables were categorized		NA
		(*c*) If relevant, consider translating estimates of relative risk into absolute risk for a meaningful time period		NA
Other analyses	17	Report other analyses done—eg analyses of subgroups and interactions, and sensitivity analyses	11-13	✓
**Discussion**				
Key results	18	Summarise key results with reference to study objectives	14	✓
Limitations	19	Discuss limitations of the study, taking into account sources of potential bias or imprecision. Discuss both direction and magnitude of any potential bias	18	✓
Interpretation	20	Give a cautious overall interpretation of results considering objectives, limitations, multiplicity of analyses, results from similar studies, and other relevant evidence	14-19	✓
Generalisability	21	Discuss the generalisability (external validity) of the study results	18	✓
**Other information**				
Funding	22	Give the source of funding and the role of the funders for the present study and, if applicable, for the original study on which the present article is based	20	✓

*Give information separately for cases and controls in case-control studies and, if applicable, for exposed and unexposed groups in cohort and cross-sectional studies.

Note: An Explanation and Elaboration article discusses each checklist item and gives methodological background and published examples of transparent reporting. The STROBE checklist is best used in conjunction with this article (freely available on the Web sites of PLoS Medicine at http://www.plosmedicine.org/, Annals of Internal Medicine at http://www.annals.org/, and Epidemiology at http://www.epidem.com/). Information on the STROBE Initiative is available at www.strobe-statement.org.

For peer review only - http://bmjopen.bmj.com/site/about/guidelines.xhtml
